# Numerical simulation and mathematical modeling for heat and mass transfer in MHD stagnation point flow of nanofluid consisting of entropy generation

**DOI:** 10.1038/s41598-023-33412-8

**Published:** 2023-04-19

**Authors:** M. Riaz Khan, V. Puneeth, Aisha M. Alqahtani, Sharifah E. Alhazmi, Sid Ahmed Ould Beinane, Meshal Shutaywi, Sayed M. Eldin, Theyab R. Alsenani

**Affiliations:** 1 Department of Mathematics, Quaid-i-Azam University, 45320, Islamabad, 44000 Pakistan; 2 Department of Computational Sciences, CHRIST University, Bengaluru, 560029 India; 3 Department of Mathematical Sciences, College of Science, Princess Nourah Bint Abdulrahman University, P. O. Box 84428, Riyadh, 11671 Saudi Arabia; 4 Mathematics Department, Al-Qunfudah University College, Umm Al-Qura University, Mecca, Saudi Arabia; 5 Mathematics Department, College of Science, Jouf University, P. O. Box 2014, Sakaka, Saudi Arabia; 6 Department of Mathematics College of Science and Arts, King Abdulaziz University, P. O. Box 344, 21911 Rabigh, Saudi Arabia; 7 Faculty of Engineering, Center of Research, Future University in Egypt, New Cairo, 11835 Egypt; 8 Department of Electrical Engineering, College of Engineering in Al-Kharj, Prince Sattam Bin Abdulaziz University, 11942 Al-Kharj, Saudi Arabia

**Keywords:** Applied mathematics, Computational science

## Abstract

The primary goal of this article is to explore the radiative stagnation point flow of nanofluid with cross-diffusion and entropy generation across a permeable curved surface. Moreover, the activation energy, Joule heating, slip condition, and viscous dissipation effects have been considered in order to achieve realistic results. The governing equations associated with the modeling of this research have been transformed into ordinary differential equations by utilizing appropriate transformation variable. The resulting system of equations was solved numerically by using Bvp4c built-in package in MATLAB. The impact of involved parameters have been graphically examined for the diverse features of velocity, temperature, and concentration profiles. Throughout the analysis, the volume fraction is assumed to be less than $5\%$ while the Prandtl number is set to be $6$. In addition, the entropy generation, friction drag, Nusselt, and Sherwood numbers have been plotted for describing the diverse physical aspects of the underlying phenomena. The major outcomes reveal that the curvature parameter reduces the velocity profile and skin friction coefficient whereas the magnetic parameter, temperature difference parameter, and radiation parameter intensify the entropy generation.

## Introduction

Incompressible viscous fluids flow over a stretching surface has captivated an extensive consideration of researchers owing to the variety of applications in engineering productions as well as scientific processes, like, metal processing industries, production of paper and glass-fiber, wire drawing, polymer, and high temperatures such as atomic power plant, gas turbine, thermal energy storage, solar power technology, and electrical power generation, etc. The dynamics of two-phase dusty fluid flow were numerically investigated by Siddiqa et al.^1^. Ahmed et al.^2^ examined the rheological behavior of incompressible viscous nanofluids considering the thermal slip. The incompressible magnetized flow of a viscous fluid through a stretching sheet was explored by Andersson et al.^3^. Pop et al.^4^ investigated the MHD flow along with the blowing phenomena induced by a stretching sheet. Gupta and Gupta^5^ numerically considered the heat and mass transfer along with suction or blowing across a stretching surface. Furthermore, Reddy et al.^6^, studied the heat and mass transfer properties of hybrid nanofluid flowing over a flat surface subjected to stretching/shrinking. Santhi et al.^7^ implemented the double stratification model and compared the steady and unsteady flow of nanofluid. Meanwhile, Basha et al.^8^ studied the ferromagnetic stagnation flow of Carreau nanofluid over a wedge and observed a declination in the velocity for the stronger magnetic field. Reddy et al.^9^ discussed the impact of thermophoresis and observed a rise in the temperature of the nanofluid for strong thermophoresis. Sreedevi and Reddy^10^ concluded that the heat conducted by the nanofluid enhances for the stronger thermophoresis and Brownian motion. Basha et al.^11^ performed a sensitivity analysis to explain the heat transport features of Eyring-Powell nanofluid flowing across a circular cylinder. Reddy et al.^12^ investigated the impact of biot number on the heat transfer characteristics of nanofluid set in motion across a vertical cone. Here is the more recent work available for the representative analysis of the nanofluid motion across a stretched surface^13–20^.

Energy loss in a flow and heat transfer development is due to irreversible procedures. Entropy generation is a prime concept in every engineering industry. Entropy plays a projecting role in thermodynamics analysis, biotechnology, statistical mechanics, fluid mechanics, fluid dynamics, and continuum physics, more recently, also in biology, etc. Entropy is related not only to the availability of energy to do work, but it is also a measure of disruption of a system as well as its surrounding. This notion was initially postulated by Ludwig Boltzmann in the 1800s using the second law of thermodynamics to calculate the entropy generation in any thermodynamic system. The latest study elucidated that the second law is a more comprehensive and effective investigation method to reduce the entropy of a system. Entropy structures are associated with a considerable number of energy-related processes including geothermic power systems and solar power systems. Originally, Bejan^21^ gave the idea of entropy in the heat transport systems and in the fluid flow systems. Sohail et al.^22^ considered the impacts of heat conductance and the thermal conductivity associated with entropy formation in magnetized fluid flow across a bi-directional stretching sheet. Zhang et al.^23^ explored the entropy study on the blood flow with magnetic Zinc-Oxide nanoparticles considering Jeffery fluid flow. Srinivasacharya and Bindu^24^ present the numerical solution of entropy optimization for the micropolar fluid motion induced by an inclined channel. Basha et al.^25^ explored the flow of a tangent hyperbolic nanofluid past a cylinder by assuming the Boussinesq approximation. Al-Mdallal et al.^26^ analyzed the entropy generation using the Keller box method for the fluid flowing across a circular cylinder. Reddy et al.^27^ examined the entropy generation process along with the heat transport features of nanofluid associated with the influence of a magnetic field. Furthermore, Basha and Sivaraj^28^ applied the collocation method to study the entropy process for the flow of ${\text{Ag}} - {\text{Fe}}_{3} {\text{O}}_{4}$—blood flowing inside a porous tube. The references^27–32^ shows some recently published work available on entropy optimization.

The process of heat and mass transportation including the influence of Dufour and Soret creates an important impact because of various applications including migration of groundwater pollutants, binary alloys solidification, melting of geosciences different components, separation of isotopes, and mixing gases. Mainly both Soret and Dufour effects can work more strongly whether the temperature is high having concentration gradients in large amount. Hayat et al.^33^ explored the transport of mass and heat under the use of these two effects with mixed convection boundary layer flow across a spongy surface in a permeable medium which is covered with viscoelastic fluid. Turkyilmazoglu and pop^34^ have discussed the heat sources effects and Soret effect on impulsively arising innumerable vertical surface with time dependent MHD radiative free convection flow. Cheng^35^ discussed the effects of Soret and Dufour on convection-free heat and mass transport from the sloppy plate in a spongy or permeable medium having the same concentration and wall temperature. The control of chemical reactions and radiation on mass transfer and heat convection over a flexible surface in the boundaries of a Darcian spongy medium with effects of Soret and Dufour phenomenon have been explored by Pal and Mondal^36^. The linear as well as the nonlinear double-diffusive convection which is saturated in an anisotropic permeable layer including Soret effect and the internal heat source has been explored by Altawallbeh et al.^37^. The additional latest work is available on the transport of heat which can be seen in the refs.^38–45^.

Based on the above studies, in the current research work, our goal is to analyze viscous nanofluid with cross-diffusion and entropy generation along with stagnation point flow across a curved surface. Moreover, the Joule heating and the activation energy have been considered in this investigation. Particularly, the target was to modify the recently published article of Revathi et al.^46^ and associate their work with stagnation point flow considering the novel terms like porosity, viscous dissipation, suction, and slip effects. Moreover, this work has been considered with the new fluid (SiO_2_–CH_3_OH), and solved numerically with the application of the bvp4c package in MATLAB. The graphical assessment has been performed to analyze various numerical results for distant values of effective parameters. In this way, the model considered here is completely different from the published work, and on the basis of the author’s knowledge, no one in the past considered such kind of investigations. The important area of application of this flow problem is manufacturing, engineering, and industrial sciences consisting of mechanical engineering, health science, civil engineering, geomechanics, bioengineering, material science, petroleum engineering, etc. The real-world examples of these applications are thermal insulation, refrigerators, filtration plants, fluidized beds, groundwater flows, heat exchangers, filtration plants, etc. Moreover, industrial and manufacturing processes like nuclear reactors, combustion, solar ponds, missile technology, furnace design, etc. are particularly based on the function of thermal radiation. Additionally, in several engineering techniques, the radiation phenomenon is used as a heat-controlling agent. Thus, the current effort will entice countless researchers owning to their extensive incredible and innovative applications which enthused us to discuss the existing work.

## Basic equations

**Figure 1:**
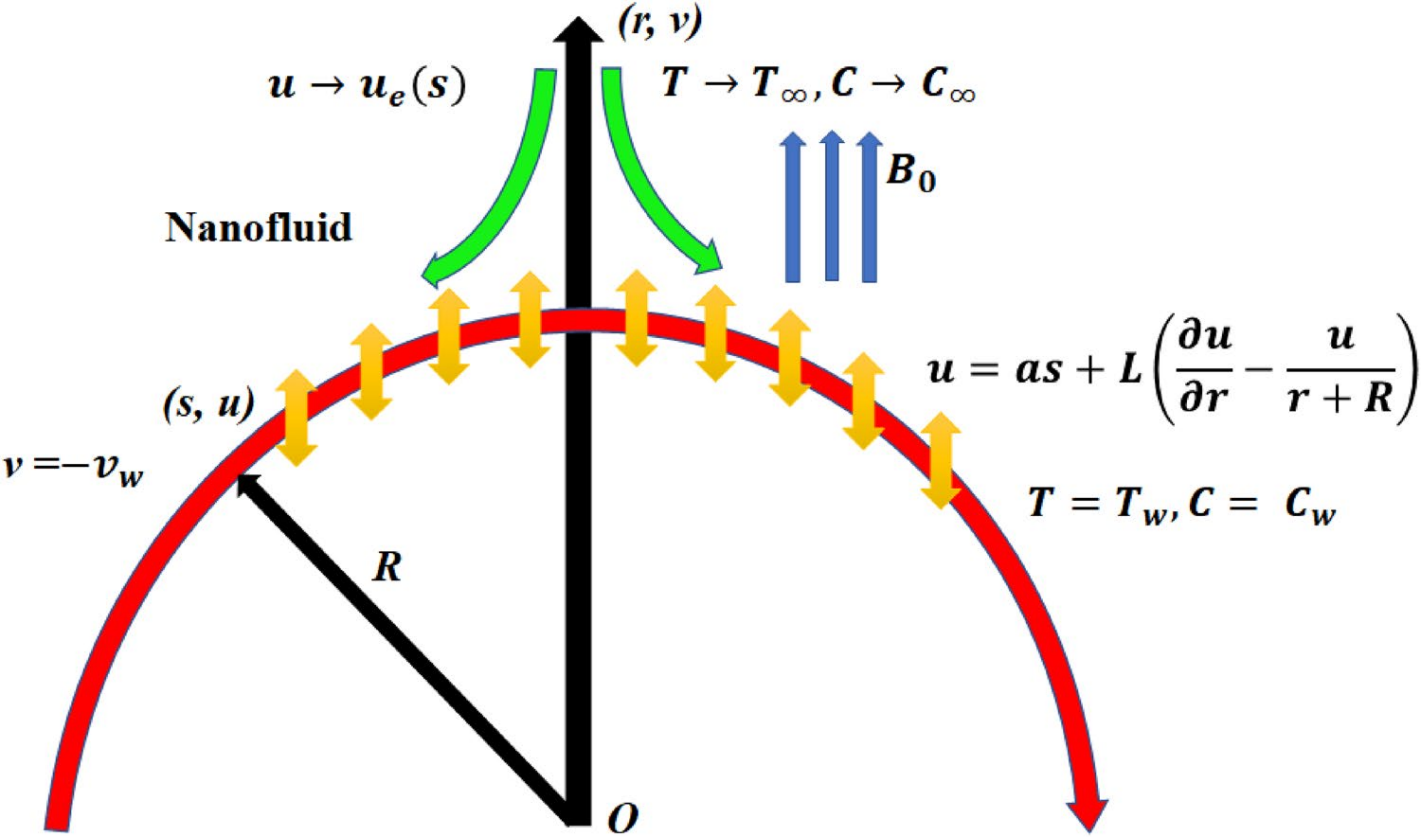
Schematic diagram.

The two-dimensional incompressible radiative stagnation point flow of a dissipative nanofluid over a permeable curved surface has been considered with the impact of Joule heating and activation energy as shown in Fig. 1. Moreover, the Soret and Dufour numbers were correspondingly considered in the mass diffusion and energy equations. The two directions $r$ and $s$ were considered correspondingly perpendicular to the surface and along the surface with the surface velocity $u = as + L\left( {\frac{\partial u}{{\partial r}} - \frac{u}{r + R}} \right)$, and with the free stream velocity $u \to u_{e} \left( s \right) = bs$, where $a > 0, a < 0$ and $a = 0$ respectively signifies the stretching, shrinking and static surface with slip length $L$. A magnetic field of intensity $B_{0}$ was fixed in the radial direction. The nanofluid was prepared by the combination of silica nano particles and the methanol base fluid. In view of these considerations, the governing boundary layer equations are stated below^47–49^.

1 $$\frac{\partial }{\partial r}\left[ {\left( {r + R} \right)v} \right] = - R\frac{\partial u}{{\partial s}},$$

2 $$\frac{1}{{\rho_{nf} }}\frac{\partial p}{{\partial r}} - \frac{{u^{2} }}{r + R} = 0,$$

3 $$\frac{1}{{\rho_{nf} }}\frac{R}{r + R}\frac{\partial p}{{\partial s}} = v_{nf} \left( {\frac{{\partial^{2} u}}{{\partial r^{2} }} + \frac{1}{r + R}\frac{\partial u}{{\partial r}} - \frac{u}{{\left( {r + R} \right)^{2} }}} \right) - v\frac{\partial u}{{\partial r}} - \frac{Ru}{{r + R}}\frac{\partial u}{{\partial s}} - \frac{uv}{{r + R}} - v_{nf} \frac{u}{{K_{p} }} - \frac{{\sigma_{nf} B_{0}^{2} }}{{\rho_{nf} }}u,$$

4 $$\begin{aligned} \left( {v\frac{\partial T}{{\partial r}} + \frac{Ru}{{r + R}}\frac{\partial T}{{\partial s}}} \right) & = \frac{{k_{nf} }}{{\left( {\rho C_{p} } \right)_{nf} }}\left( {\frac{{\partial^{2} T}}{{\partial r^{2} }} + \frac{1}{r + R}\frac{\partial T}{{\partial r}}} \right) + \frac{{\sigma_{nf} }}{{\left( {\rho C_{p} } \right)_{nf} }}B_{0}^{2} u^{2} \\ & \quad + \frac{1}{{\left( {\rho C_{p} } \right)_{nf} }}\left( {\frac{{\partial^{2} T}}{{\partial r^{2} }} + \frac{1}{r + R}\frac{\partial T}{{\partial r}}} \right)\frac{{16\sigma^{*} T_{\infty }^{3} }}{{3k^{*} }} + \frac{{\mu_{nf} }}{{\left( {\rho C_{p} } \right)_{nf} }}\left( {\frac{\partial u}{{\partial r}} - \frac{u}{r + R}} \right)^{2} \\ & \quad + \frac{1}{{\left( {\rho C_{p} } \right)_{nf} }}\frac{{D_{m} k_{T} }}{{c_{s} }}\left( {\frac{{\partial^{2} C}}{{\partial r^{2} }} + \frac{1}{r + R}\frac{\partial C}{{\partial r}}} \right), \\ \end{aligned}$$

5 $$\begin{aligned} \left( {v\frac{\partial C}{{\partial r}} + \frac{Ru}{{r + R}}\frac{\partial C}{{\partial s}}} \right) & = D_{m} \left( {\frac{{\partial^{2} C}}{{\partial r^{2} }} + \frac{1}{r + R}\frac{\partial C}{{\partial r}}} \right) + \frac{{D_{m} k_{T} }}{{T_{m} }}\left( {\frac{{\partial^{2} T}}{{\partial r^{2} }} + \frac{1}{r + R}\frac{\partial T}{{\partial r}}} \right) \\ & \quad - K_{r}^{2} \left( {\frac{T}{{T_{\infty } }}} \right)^{n} Exp\left( { - \frac{{E_{a} }}{{K_{1} T}}} \right)\left( {C - C_{\infty } } \right). \\ \end{aligned}$$

The related boundary conditions are specified as

6 $$\left. {\begin{array}{*{20}c} { u = as + L\left( {\frac{\partial u}{{\partial r}} - \frac{u}{r + R}} \right),v = - v_{w} , T = T_{w} , C = C_{w} , at\, r = 0,} \\ {u \to u_{e} \left( s \right) = bs, \frac{\partial u}{{\partial r}} \to 0, T \to T_{\infty } , C \to C_{\infty }\, as \, r \to \infty .} \\ \end{array} } \right\}$$

The thermophysical characteristics of the Silica (SiO_2_) nanoparticles and methanol (CH_3_OH) base fluid is specified in Tables 1 and 2.

**Table 1:** The thermophysical properties of nanofluid.

Properties	Nanofluid
Density	$\rho_{nf} = \varphi \rho_{s} + \left( {1 - \varphi } \right)\rho_{f}$
Electrical conductivity	$\frac{{\sigma_{nf} }}{{\sigma_{f} }} = 1 + \frac{{3\left( {\frac{{\sigma_{s} }}{{\sigma_{f} }} - 1} \right)\varphi }}{{\left( {\frac{{\sigma_{s} }}{{\sigma_{f} }} + 2} \right) - \left( {\frac{{\sigma_{s} }}{{\sigma_{f} }} - 1} \right)\varphi }}$
Heat capacity	$\left( {\rho C_{p} } \right)_{nf} = \varphi \left( {\rho C_{p} } \right)_{s} + \left( {1 - \varphi } \right)\left( {\rho C_{p} } \right)_{f}$
Viscosity	$\mu_{nf} = \frac{{\mu_{f} }}{{\left( {1 - \varphi } \right)^{2.5} }}$
Thermal diffusivity	$\alpha_{nf} = \frac{{k_{nf} }}{{\left( {\rho C_{p} } \right)_{nf} }}$
Thermal conductivity	$\frac{{k_{nf} }}{{k_{f} }} = \frac{{\left( {\frac{{k_{s} }}{{k_{f} }} + 2} \right) - 2\varphi \left( {1 - \frac{{k_{s} }}{{k_{f} }}} \right)}}{{\left( {\frac{{k_{s} }}{{k_{f} }} + 2} \right) + \varphi \left( {1 - \frac{{k_{s} }}{{k_{f} }}} \right)}}$

**Table 2:** Thermophysical features of the Silica (SiO_2_) nanoparticles and the methanol (CH_3_OH) base fluid.

Thermophysical properties	Methanol (f) ( CH_3_OH)	Silica (s) ( SiO_2_)
$C_{p}$ (J/kgK)	2545	703
$\rho$ (Kg/m^3^)	792	2200
$k$ (W/mK)	0.2035	1.38
$\sigma$ (S/m)	$0.5 \times 10^{ - 6}$	$10^{ - 25}$

It is possible to convert Eqs. (1–6) into a non-dimensional structure by introducing the following dimensionless transformations.

7 $$\left. {\begin{array}{*{20}c} {u = bsf^{\prime } \left( \eta \right), \eta = \sqrt {\frac{b}{{v_{f} }}} r, v = - \frac{R}{r + R}\sqrt {bv_{f} } f\left( \eta \right) , p = \rho_{f} u_{e}^{2} P\left( \eta \right)} \\ {T = \theta \left( \eta \right)\left( {T_{w} - T_{\infty } } \right) + T_{\infty } , C = \phi \left( \eta \right)\left( {C_{w} - C_{\infty } } \right) + C_{\infty } } \\ \end{array} } \right\}.$$

Thus, the resulting non-dimensional equation assumes the following structure.

8 $$\frac{{\rho_{f} }}{{\rho_{nf} }}\frac{\partial P}{{\partial \eta }} = \frac{1}{\eta + K}f^{{\prime^{2} }} ,$$

9 $$\begin{aligned} \frac{{\rho_{f} }}{{\rho_{nf} }}\frac{2K}{{\eta + K}}P & = \frac{{v_{nf} }}{{v_{f} }}\left( {f^{\prime \prime \prime } - \frac{1}{{\left( {\eta + K} \right)^{2} }}f^{\prime } + \frac{1}{\eta + K}f^{\prime \prime } } \right) - \frac{K}{\eta + K}\left( {f^{\prime } } \right)^{2} + \frac{K}{\eta + K}ff^{\prime \prime } \\ & \quad + \frac{K}{{\left( {\eta + K} \right)^{2} }}ff^{\prime } - \delta f^{\prime } - M^{2} \frac{{\sigma_{nf} }}{{\sigma_{f} }}\frac{{\rho_{f} }}{{\rho_{nf} }}f^{\prime } , \\ \end{aligned}$$

10 $$\begin{aligned} & \frac{1}{{{\text{Pr}}}}\frac{{k_{nf} }}{{k_{f} }}\frac{{\left( {\rho C_{p} } \right)_{f} }}{{\left( {\rho C_{p} } \right)_{nf} }}\left( {1 + \frac{4}{3}Rd} \right)\left( {\theta^{\prime \prime } + \frac{1}{\eta + K}\theta^{\prime } } \right) + \frac{K}{\eta + K}f\theta^{\prime } + \frac{{\mu_{nf} }}{{\mu_{f} }}\frac{{\left( {\rho C_{p} } \right)_{f} }}{{\left( {\rho C_{p} } \right)_{nf} }}E_{c} \left( {f^{\prime \prime } - \frac{{f^{\prime } }}{\eta + K}} \right)^{2} \\ & + M^{2} E_{c} \frac{{\left( {\rho C_{p} } \right)_{f} }}{{\left( {\rho C_{p} } \right)_{nf} }}\frac{{\sigma_{nf} }}{{\sigma_{f} }}\left( {f^{\prime } } \right)^{2} + {\text{Du}}\left( {\phi^{\prime \prime } + \frac{1}{\eta + K}\phi^{\prime } } \right) = 0, \\ \end{aligned}$$

11 $$\phi^{\prime \prime } + \frac{1}{\eta + K}\phi^{\prime } + {\text{Sc}}\left\{ {\frac{K}{\eta + K}f\phi^{\prime } + Sr\left( {\theta^{\prime \prime } + \frac{1}{\eta + K}\theta^{\prime } } \right) - \tau \left( {1 + \omega \theta } \right)^{n} Exp\left( { - \frac{{E_{1} }}{1 + \omega \theta }} \right)} \right\} = 0,$$

Based on Eq. (8), we can eliminate the pressure $P$ from Eq. (9). Thus, the combination of Eqs. (8 and 9) can be written as.

12 $$\begin{aligned} & f^{iv} + \frac{2}{\eta + K}f^{\prime \prime \prime } + \frac{1}{{\left( {\eta + K} \right)^{3} }}f^{\prime } - \frac{1}{{\left( {\eta + K} \right)^{2} }}f^{\prime \prime } + \frac{{v_{f} }}{{v_{nf} }}\left[ {\frac{K}{\eta + K}\left( {ff^{\prime \prime \prime } - f^{\prime } f^{\prime \prime } } \right) + \frac{K}{{\left( {\eta + K} \right)^{2} }}\left( {ff^{\prime \prime } - f^{{\prime^{2} }} } \right)} \right. \\ & \quad \left. { - \frac{K}{{\left( {\eta + K} \right)^{3} }}ff^{\prime } - \delta f^{\prime \prime } - M^{2} \frac{{\sigma_{nf} }}{{\sigma_{f} }}\frac{{\rho_{f} }}{{\rho_{nf} }}\left( {f^{\prime \prime } + \frac{1}{\eta + K}f^{\prime } } \right)} \right] = 0. \\ \end{aligned}$$

By re-arranging Eqs. (10) and (11), we get.

13 $$\begin{aligned} & \left\{ {\frac{1}{{{\text{Pr}}}}\frac{{k_{nf} }}{{k_{f} }}\frac{{\left( {\rho C_{p} } \right)_{f} }}{{\left( {\rho C_{p} } \right)_{nf} }}\left( {1 + \frac{4}{3}Rd} \right) - {\text{Sr\, ScDu}}} \right\}\theta^{\prime \prime } + \left\{ {\frac{1}{Pr}\frac{{k_{nf} }}{{k_{f} }}\frac{{\left( {\rho C_{p} } \right)_{f} }}{{\left( {\rho C_{p} } \right)_{nf} }}\left( {1 + \frac{4}{3}Rd} \right)\frac{1}{\eta + K}} \right. \\ & \quad \left. { - {\text{Sr}} \,{\text{ScDu}}\frac{1}{\eta + K} + \frac{K}{\eta + K}f} \right\}\theta^{\prime } + {\text{DuSc}} \frac{K}{\eta + K}f\phi^{\prime } + E_{c} \frac{{\mu_{nf} }}{{\mu_{f} }}\frac{{\left( {\rho C_{p} } \right)_{f} }}{{\left( {\rho C_{p} } \right)_{nf} }}\left( {f^{\prime \prime } - \frac{{f^{\prime } }}{\eta + K}} \right)^{2} \\ & \quad + M^{2} E_{c} \frac{{\left( {\rho C_{p} } \right)_{f} }}{{\left( {\rho C_{p} } \right)_{nf} }}\frac{{\sigma_{nf} }}{{\sigma_{f} }}\left( {f^{\prime } } \right)^{2} + \tau {\text{Du}}\left( {1 + \omega \theta } \right)^{n} {\text{Sc}} \, Exp\left( { - \frac{{E_{1} }}{1 + \omega \theta }} \right) = 0, \\ \end{aligned}$$

14 $$\phi^{\prime \prime } + \frac{1}{\eta + K}\phi^{\prime } + {\text{Sc}}\left\{ {\frac{K}{\eta + K}f\phi^{\prime } + {\text{Sr}}\left( {\theta^{\prime \prime } + \frac{1}{\eta + K}\theta^{\prime } } \right) - \tau \left( {1 + \omega \theta } \right)^{n} Exp\left( { - \frac{{E_{1} }}{1 + \omega \theta }} \right)} \right\} = 0,$$

The boundary conditions (6) are transformed into following pattern.

15 $$\left. {\begin{array}{*{20}c} {f\left( 0 \right) = S, f^{\prime } \left( 0 \right) = \lambda + \epsilon \left\{ {\frac{{f^{\prime } \left( 0 \right)}}{K} + f^{^{\prime\prime}} \left( 0 \right)} \right\}, \theta \left( \eta \right) = 1, \phi \left( \eta \right) = 1} \\ {f^{\prime } \left( \eta \right) = 1, f^{\prime \prime } \left( \eta \right) = 0, \theta \left( \eta \right) = 0, \phi \left( \eta \right) = 0 \, as \, \eta \to \infty .} \\ \end{array} } \right\}.$$

The dimensionless parameters arising from Eqs. (12–15) are defined below.

16 $$\left. \begin{aligned} & K = R\sqrt {\frac{b}{{v_{f} }}} , \delta = \frac{{\nu_{f} }}{{bK_{p} }}, M^{2} = \frac{{\sigma_{f} B_{0}^{2} }}{{b\rho_{f} }}, {\text{Pr}} = \frac{{\nu_{f} }}{{\alpha_{f} }},E_{c} = \frac{{u^{2} }}{{C_{p} \Delta T}} = \frac{{b^{2} s^{2} }}{{C_{p} \left( {T_{w} - T_{\infty } } \right)}}, \\ & Rd = \frac{{4\sigma^{*} T_{\infty }^{3} }}{{k_{f} k^{*} }}, {\text{Du}} = \frac{{D_{m} k_{T} \left( {C_{w} - C_{\infty } } \right)}}{{c_{s} \left( {\mu C_{p} } \right)_{f} \left( {T_{w} - T_{\infty } } \right)}}, {\text{Sc}} = \frac{{\nu_{f} }}{{D_{m} }},{\text{Sr}} = \frac{{D_{m} k_{T} \left( {C_{w} - C_{\infty } } \right)}}{{T_{m} \nu \left( {T_{w} - T_{\infty } } \right)}}, \\ & \tau = \frac{{K_{r}^{2} }}{b}, \omega = \frac{{T_{w} - T_{\infty } }}{{T_{\infty } }}, E_{1} = \frac{{E_{a} }}{{k_{1} T_{\infty } }}, \epsilon = L\sqrt {\frac{b}{{v_{f} }}} , \lambda = \frac{a}{b}. \\ \end{aligned} \right\}$$

In addition, it must be noted that by presuming $K \to \infty$, the classical problem of the flat sheet is acquired. In this way, Eq. (12) is changed into Eq. (17).

17 $$\frac{{v_{nf} }}{{v_{f} }}f^{^{\prime}v} + ff^{\prime \prime \prime } - f^{\prime } f^{\prime \prime } - \delta f^{\prime \prime } - M^{2} \frac{{\sigma_{nf} }}{{\sigma_{f} }}\frac{{\rho_{f} }}{{\rho_{nf} }}f^{\prime \prime } = 0,$$

The integration of Eq. (17), prior to the utilization of condition (15) at $\eta \to \infty$ yield the following.

18 $$\frac{{v_{nf} }}{{v_{f} }}f^{\prime \prime \prime } + ff^{\prime \prime } - f^{{1^{2} }} - \delta f^{\prime } - M^{2} \frac{{\sigma_{nf} }}{{\sigma_{f} }}\frac{{\rho_{f} }}{{\rho_{nf} }}f^{\prime \prime } + M^{2} + \delta + 1 = 0$$

For the similar condition ($K \to \infty$), Eqs. (13) and (14) reduces to Eqs. (19) and (20).

19 $$\begin{aligned} & \frac{1}{{{\text{Pr}}}}\frac{{k_{nf} }}{{k_{f} }}\frac{{\left( {\rho C_{p} } \right)_{f} }}{{\left( {\rho C_{p} } \right)_{nf} }}\left( {1 + \frac{4}{3}Rd} \right)\theta^{\prime \prime } + {\text{Sr}}\, {\text{Sc}}\, {\text{Du}}\, \theta^{\prime \prime } + \left\{ {\theta^{\prime } - {\text{Sc\,Du}}\, \phi^{\prime } } \right\}f \\ & \quad + \frac{{\mu_{nf} }}{{\mu_{f} }}\frac{{\left( {\rho C_{p} } \right)_{f} }}{{\left( {\rho C_{p} } \right)_{nf} }}E_{c} \left( {f^{\prime \prime } } \right)^{2} + M^{2} E_{c} \frac{{\left( {\rho C_{p} } \right)_{f} }}{{\left( {\rho C_{p} } \right)_{nf} }}\frac{{\sigma_{nf} }}{{\sigma_{f} }}\left( {f^{\prime}} \right)^{2} \\ & \quad + \tau {\text{Du}}\left( {1 + \omega \theta } \right)^{n} {\text{Sc}}\, Exp\left( { - \frac{{E_{1} }}{1 + \omega \theta }} \right) = 0, \\ \end{aligned}$$

20 $$\phi^{\prime \prime } + {\text{Sc}}\left\{ {{\text{Sr}}\theta^{\prime \prime } - \alpha \left( {1 + \sigma \theta } \right)^{n} Exp\left( { - \frac{{E_{1} }}{1 + \sigma \theta }} \right)} \right\} = 0.$$

and conditions (15) reduces to.

21 $$\left. {\begin{array}{*{20}c} {f\left( 0 \right) = S, f^{\prime } \left( 0 \right) = \lambda + \epsilon f^{\prime \prime } \left( 0 \right), \theta \left( \eta \right) = 1, \, \phi \left( \eta \right) = 1} \\ {f^{\prime } \left( \eta \right) = 1, f^{\prime \prime } \left( \eta \right) = 0, \theta \left( \eta \right) = 0, \phi \left( \eta \right) = 0 \, as\, \eta \to \infty .} \\ \end{array} } \right\}.$$

The important physical quantities about the engineering interest associated with the present study are the local Nusselt number $\left( {{\text{Nu}}_{s} } \right)$, friction drag $\left( {C_{fs} } \right)$ and Sherwood number (${\text{Sh}}_{s}$), which could be defined as

22 $$C_{fs} = \frac{{\tau_{rs} }}{{\rho_{f} u_{w}^{2} \left( s \right)}}, {\text{Nu}}_{s} = \frac{{sq_{w} }}{{k_{f} \left( {T_{w} - T_{\infty } } \right)}},{\text{Sh}}_{s} = \frac{{sj_{w} }}{{D_{m} \left( {C_{w} - C_{\infty } } \right)}}.$$

where

23 $$\tau_{rs} = \mu_{nf} \left. {\left( {\frac{\partial u}{{\partial r}} - \frac{u}{r + R}} \right)} \right|_{r = 0} , q_{w} = - k_{nf} \left( {1 + \frac{{16\sigma^{*} T_{\infty }^{3} }}{{3k_{f} k^{*} }}\frac{{k_{f} }}{{k_{nf} }}} \right)\left. {\frac{\partial T}{{\partial r}}} \right|_{r = 0} , j_{w} = - D_{m} \left. {\frac{\partial C}{{\partial r}}} \right|_{r = 0} .$$

$\tau_{w} , q_{w}$ and $j_{w}$ correspondingly denotes the wall shear stress, heat flux as well as the mass flux.

Making use of Eq. (7) in system (22) yield the subsequent non-dimensional system.

24 $$\left. \begin{aligned} & \left( {{\text{Re}}_{s} } \right)^{\frac{1}{2}} { }C_{fs} = \frac{{\mu_{nf} }}{{\mu_{f} }}\left\{ {f^{\prime \prime } \left( 0 \right) - \frac{{f^{\prime } \left( 0 \right)}}{K}} \right\} \\ & \left( {{\text{Re}}_{s} } \right)^{ - 1/2} { }Nu_{s} { } = - \frac{{k_{nf} }}{{k_{f} }}\left( {1 + \frac{4}{3}Rd} \right)\theta^{\prime } \left( 0 \right) \\ & \left( {{\text{Re}}_{s} } \right)^{ - 1/2} { }Sh_{s} { } = - \phi \left( 0 \right) \\ \end{aligned} \right\}.$$

where $Re_{s} { } = \frac{{bs^{2} { }}}{{\nu_{f} }}{ }$ refer to the Reynolds number.

## Entropy generation modeling

The definition of entropy generation is given by

25 $$S_{gen} = \frac{{k_{nf} }}{{T_{\infty }^{2} }}\left\{ {1 + \frac{{16\sigma^{*} T_{\infty }^{3} }}{{3k_{f} k^{*} }}} \right\}\left( {\frac{\partial T}{{\partial r}}} \right)^{2} + \frac{{\mu_{nf} }}{{T_{\infty } }}\left( {\frac{\partial u}{{\partial r}} + \frac{u}{R + r}} \right)^{2} + \frac{{\sigma_{nf} B_{0}^{2} }}{{T_{\infty } }}u^{2} + \frac{{RD_{m} }}{{C_{\infty } }}\left( {\frac{\partial C}{{\partial r}}} \right)^{2} + \frac{{RD_{m} }}{{T_{\infty } }}\left( {\frac{\partial T}{{\partial r}}\frac{\partial C}{{\partial r}}} \right) + \frac{{\mu_{nf} }}{{T_{\infty } }}\frac{{u^{2} }}{{K_{p} }}.$$

In this equation, the primary, secondary and tertiary term at the right side correspondingly stands for the irreversibility of heat transfer, viscous dissipation, and Joule heating. The association of fourth term with fifth term stands for the mass transfer irreversibility as well as the last term stands for the porous medium irreversibility. Note that, $R$ is the constant of universal gas.

The use of Eq. (7), yield the dimensionless form of Eq. (25), which may be written as

26 $$N_{G} = \left( {1 + \frac{4}{3}Rd} \right)\omega \theta^{{\prime^{2} }} + B_{r} \left( {f^{\prime \prime } + \frac{1}{\eta + K}f^{\prime } } \right)^{2} + MB_{r} f^{{\prime^{2} }} + H\frac{{\omega_{1} }}{\omega }\phi^{{\prime^{2} }} + H\theta^{\prime } \phi^{\prime } + \delta B_{r} f^{{\prime^{2} }} ,$$

where the parameters $N_{G} , B_{r} , H , \omega$ and $\omega_{1}$ are defined below.

27 $$N_{G} = \frac{{T_{\infty } \nu_{f} S_{G} }}{{bk_{nf} {\Delta }T}}, B_{r} = \frac{{\mu_{nf} b^{2} s^{2} }}{{k_{nf} {\Delta }T}}, H = \frac{{RD_{m} \left( {C_{w} - C_{\infty } } \right)}}{{k_{nf} }}, \omega = \frac{{T_{w} - T_{\infty } }}{{T_{\infty } }} = \frac{{{\Delta }T}}{{T_{\infty } }}, \omega_{1} = \frac{{C_{w} - C_{\infty } }}{{C_{\infty } }} = \frac{{{\Delta C}}}{{C_{\infty } }} ,$$

The non-dimensional Bejan number could be defined as

28 $$Be = \frac{{\text{Entropy generation associated to heat and mass transfer}}}{{\text{Total entropy generation}}},$$

This implies that.

29 $$Be = \frac{{\left( {1 + \frac{4}{3}Rd} \right)\omega \theta^{{\prime}{2}} + H\frac{{\omega_{1} }}{\omega }\phi^{{\prime}{2}} + H\theta^{\prime}\phi^{\prime}}}{{\left( {1 + \frac{4}{3}Rd} \right)\omega \theta^{{\prime}{2}} + B_{r} \left( {f^{\prime\prime} + \frac{1}{\eta + K}f^{\prime}} \right)^{2} + MB_{r} f^{{\prime}{2}} + H\frac{{\omega_{1} }}{\omega }\phi^{{\prime}{2}} + H\theta^{\prime}\phi^{\prime} + \delta B_{r} f^{{\prime}{2}} }},$$

## Solution method

Here, we choose to implement the numerical approach in order to address the current flow problem. So, the differential equations must first be expressed in a system of first order ODEs before they can be solved by any differential equation solver. As a result, by including the following factors.

$$f\left( \eta \right) = y\left( 1 \right), f^{\prime } \left( \eta \right) = y\left( 2 \right), f^{\prime \prime } \left( \eta \right) = y\left( 3 \right), f^{\prime \prime \prime } \left( \eta \right) = y\left( 4 \right), f^{^{\prime}v} \left( \eta \right) = yy_{1} ,$$

$$\theta \left( \eta \right) = y\left( 5 \right), \theta^{\prime } \left( \eta \right) = y\left( 6 \right), \theta^{\prime \prime } \left( \eta \right) = yy_{2}$$

$$\phi \left( \eta \right) = y\left( 7 \right), \phi^{\prime } \left( \eta \right) = y\left( 8 \right), \phi^{\prime \prime } \left( \eta \right) = yy_{3}$$

we could rewrite the resulting Eqs. (12–14) as

30 $$\begin{aligned} yy_{1} & = - \frac{2}{x + K}y\left( 4 \right) + \frac{1}{{\left( {x + K} \right)^{2} }}y\left( 3 \right) - \frac{1}{{\left( {\eta + K} \right)^{3} }}y\left( 2 \right) - \frac{B}{A}\left[ {\frac{K}{x + K}\left( {y\left( 1 \right)y\left( 4 \right) - y\left( 3 \right)y\left( 2 \right)} \right)} \right. \\ & \quad + \frac{K}{{\left( {x + K} \right)^{2} }}\left( {y\left( 3 \right)y\left( 1 \right) - \left( {y\left( 2 \right)} \right)^{2} } \right) - \frac{K}{{\left( {x + K} \right)^{3} }}y\left( 1 \right)y\left( 2 \right) - \delta y\left( 3 \right) - \\ & \quad \left. {M^{2} \frac{C}{B}\left( {y\left( 3 \right) + \frac{1}{x + K}y\left( 2 \right)} \right)} \right], \\ \end{aligned}$$

31 $$\begin{aligned} yy_{2} & = - \frac{Pr}{{\left\{ { \frac{D}{E}\left( {1 + R} \right) - {\text{Sc}}\, {\text{Sr}}\, {\text{Du}}} \right\}}}\left[ {\left\{ {\frac{1}{{{\text{Pr}}}}\frac{D}{E}\left( {1 + R} \right)\frac{1}{\eta + K} - {\text{Sr}} \,{\text{Sc\,Du}}\frac{1}{\eta + K} + \frac{K}{\eta + K}y\left( 1 \right)} \right\}y\left( 6 \right)} \right. \\ & \quad + {\text{Du}}\, {\text{Sc}}\frac{K}{\eta + K}y\left( 1 \right)y\left( 8 \right) + E_{c} \frac{A}{E}\left( {y\left( 3 \right) - \frac{1}{\eta + K}y\left( 2 \right)} \right)^{2} + M^{2} E_{c} \frac{C}{E}\left( {y\left( 2 \right)} \right)^{2} \\ & \quad \left. {\tau {\text{Du}}\left( {1 + \omega y\left( 5 \right)} \right)^{n} {\text{Sc}}\, Exp\left( { - \frac{{E_{1} }}{1 + \omega y\left( 5 \right)}} \right)} \right], \\ \end{aligned}$$

32 $$yy_{3} = - \frac{1}{x + K}y\left( 8 \right) - {\text{Sc}}\left\{ {\frac{K}{x + K}y\left( 1 \right)y\left( 8 \right) + {\text{Sr}}\left( {yy_{2} + \frac{1}{x + K}y\left( 6 \right)} \right) - \tau \left( {1 + \omega y\left( 5 \right)} \right)^{n} Exp\left( { - \frac{{E_{1} }}{1 + \omega y\left( 5 \right)}} \right)} \right\}.$$

where

33 $$\begin{aligned} & \\ \left. \begin{aligned} \frac{{v_{f} }}{{v_{{nf}} }} = \frac{{\mu _{f} }}{{\mu _{{nf}} }}\frac{{\rho _{{nf}} }}{{\rho _{f} }} = \left( {1 - \varphi } \right)^{{2.5}} \left( {1 - \varphi + \varphi \frac{{\rho _{s} }}{{\rho _{f} }}} \right) = \frac{B}{A}~\;where~\;A = \frac{{\mu _{{nf}} }}{{\mu _{f} }} = \frac{1}{{\left( {1 - \varphi } \right)^{{2.5}} }} \\ & \frac{{\sigma _{{nf}} }}{{\sigma _{f} }}\frac{{\rho _{f} }}{{\rho _{{nf}} }} = \frac{{\text{B}}}{{\text{C}}}~\;where~\;C = \frac{{\sigma _{{nf}} }}{{\sigma _{f} }} = 1 + \frac{{3\left( {\frac{{\sigma _{s} }}{{\sigma _{f} }} - 1} \right)\varphi }}{{\left( {\frac{{\sigma _{s} }}{{\sigma _{f} }} + 2} \right) - \left( {\frac{{\sigma _{s} }}{{\sigma _{f} }} - 1} \right)\varphi }},D = ~\frac{{k_{{nf}} }}{{k_{f} }} = \frac{{\left( {\frac{{k_{s} }}{{k_{f} }} + 2} \right) - 2\varphi \left( {1 - \frac{{k_{s} }}{{k_{f} }}} \right)}}{{\left( {\frac{{k_{s} }}{{k_{f} }} + 2} \right) + \varphi \left( {1 - \frac{{k_{s} }}{{k_{f} }}} \right)}} \\ & E = \frac{{\left( {\rho C_{p} } \right)_{{nf}} }}{{\left( {\rho C_{p} } \right)_{f} }} = 1 - \varphi + \varphi \frac{{\left( {\rho C_{p} } \right)_{s} }}{{\left( {\rho C_{p} } \right)_{f} }},~R = \frac{4}{3}Rd \\ \end{aligned} \right\} \\ \end{aligned}$$

The non-dimensional boundary conditions (15) can be expressed similarly as

34 $$\left. {\begin{array}{*{20}c} {y\left( 2 \right) - \lambda - \epsilon \left\{ {y\left( 3 \right) + \frac{1}{K}y\left( 2 \right)} \right\}, y\left( 1 \right) - S, y\left( 5 \right) = 1, y\left( 7 \right) = 1 \;at\; \eta = 0} \\ {y\left( 2 \right) = 1, y\left( 3 \right) = 0, y\left( 5 \right) = 0, y\left( 7 \right) \;as\; \eta \to \infty } \\ \end{array} } \right\},$$

The three separate kinds of data are required by the bvp4c solver for boundary value problems: the equation to be solved, the correlated boundary conditions, and the initial guess utilized to arrive at the answer. Here, the relative tolerance was considered to be $10^{ - 10}$ and the integration interval was set at zero to five around the mesh point 70. The graphic below contains a sketch of the entire technique.
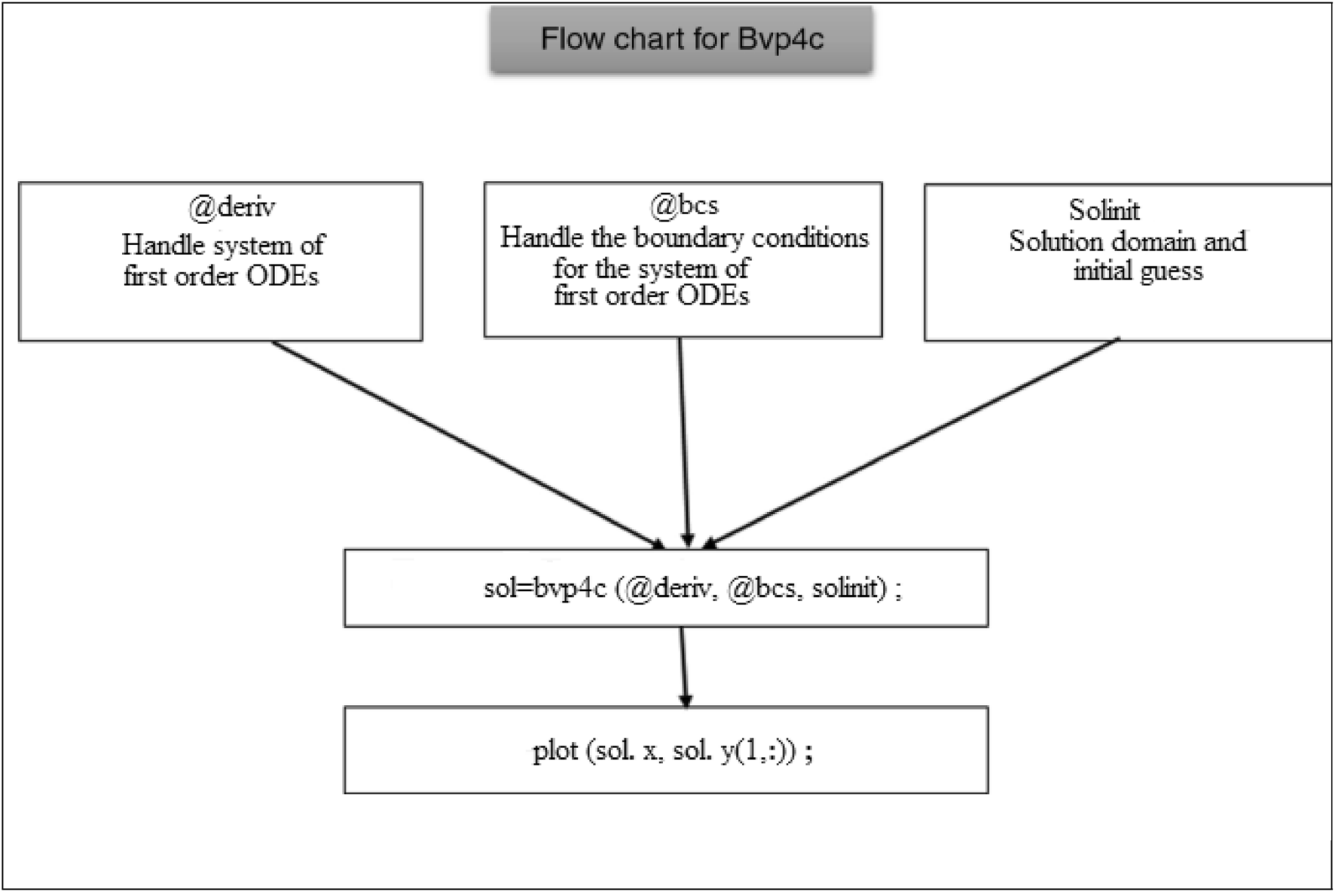


## Results and discussion

**Figure 2:**
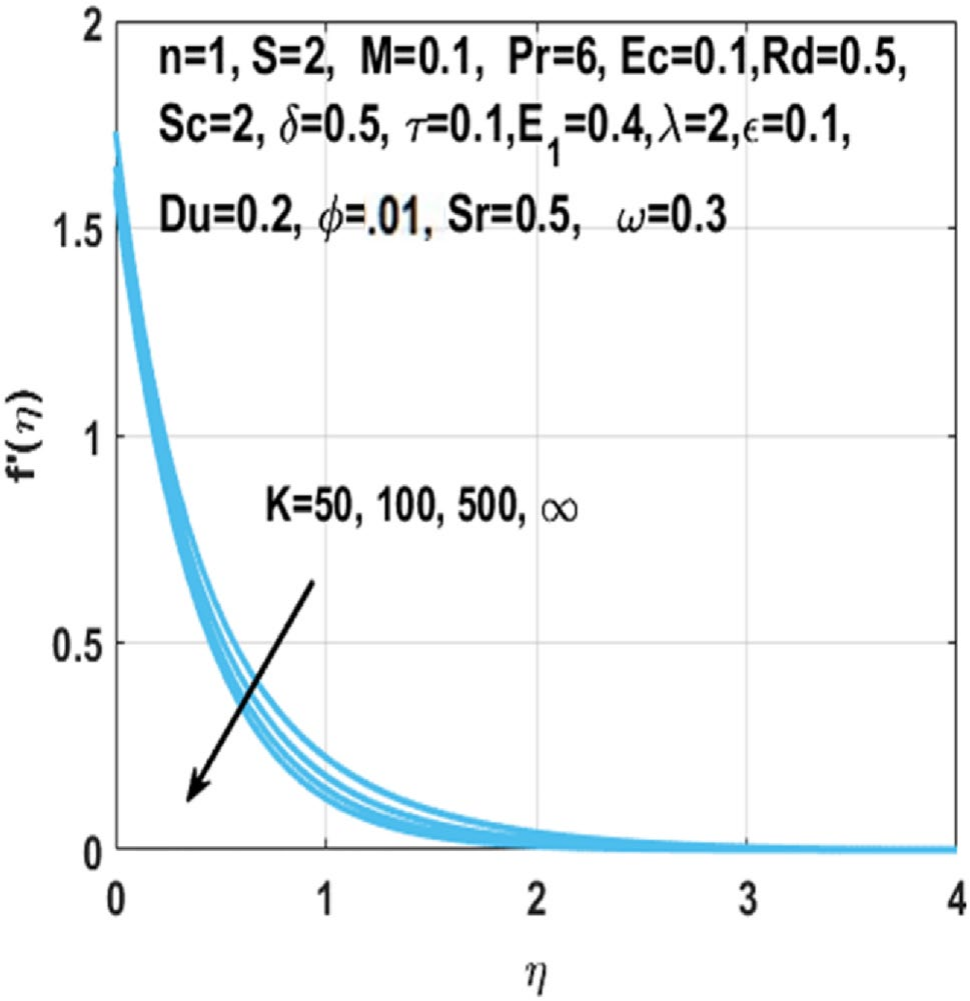
Impact of $K$ on $f^{\prime } \left( \eta \right)$.

**Figure 3:**
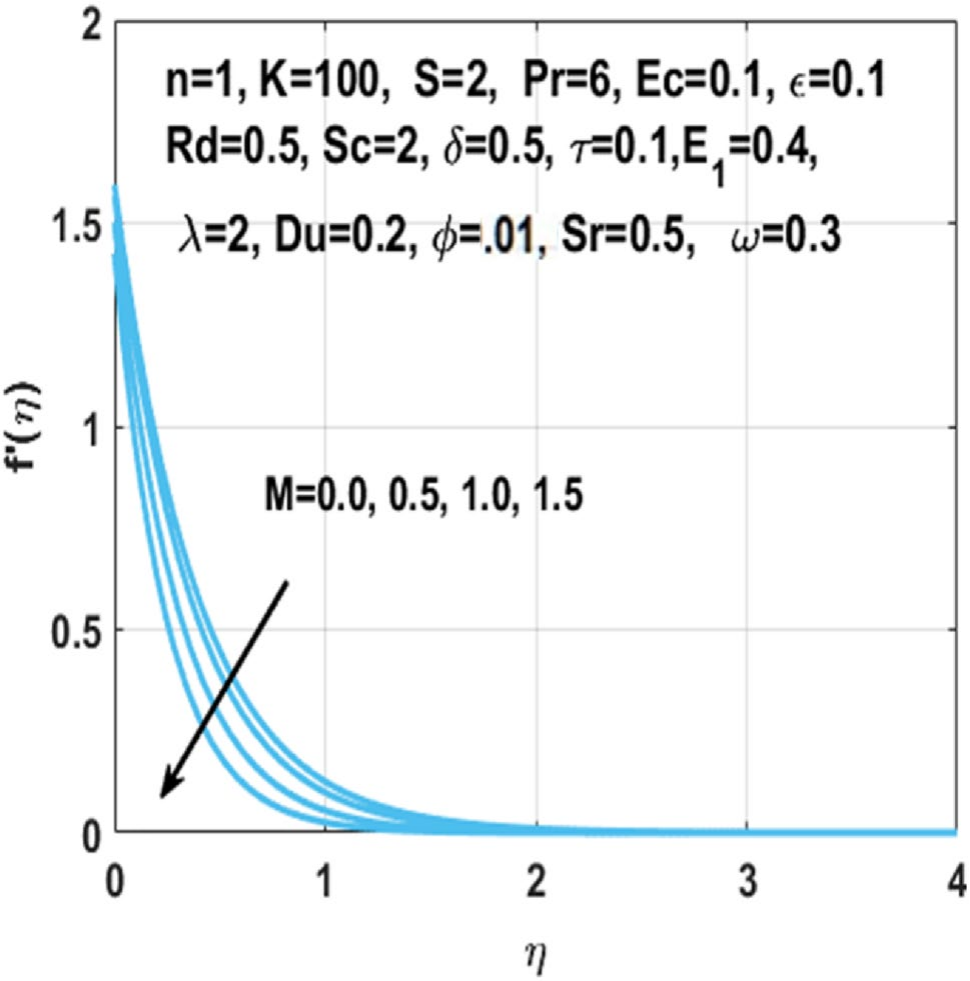
Impact of $M$ on $f^{\prime } \left( \eta \right)$.

**Figure 4:**
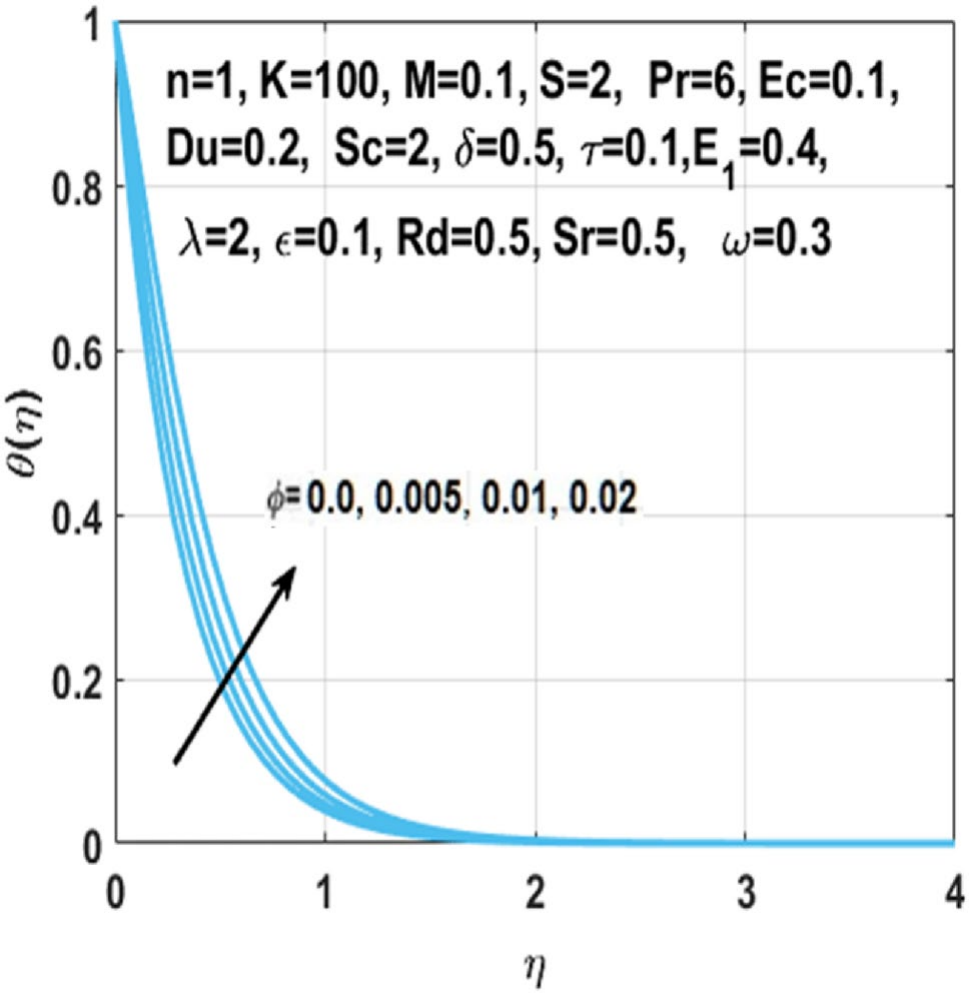
Impact of $\phi$ on $\theta \left( \eta \right)$.

**Figure 5:**
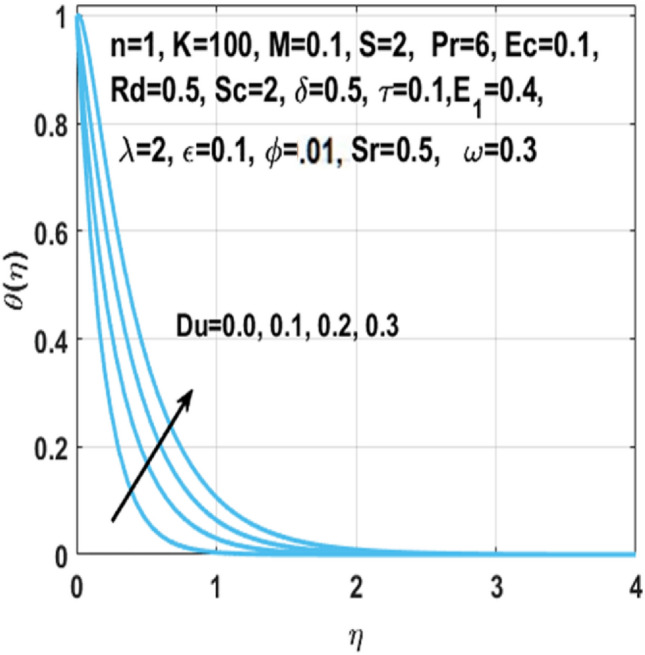
Impact of $Du$ on $\theta \left( \eta \right)$.

**Figure 6:**
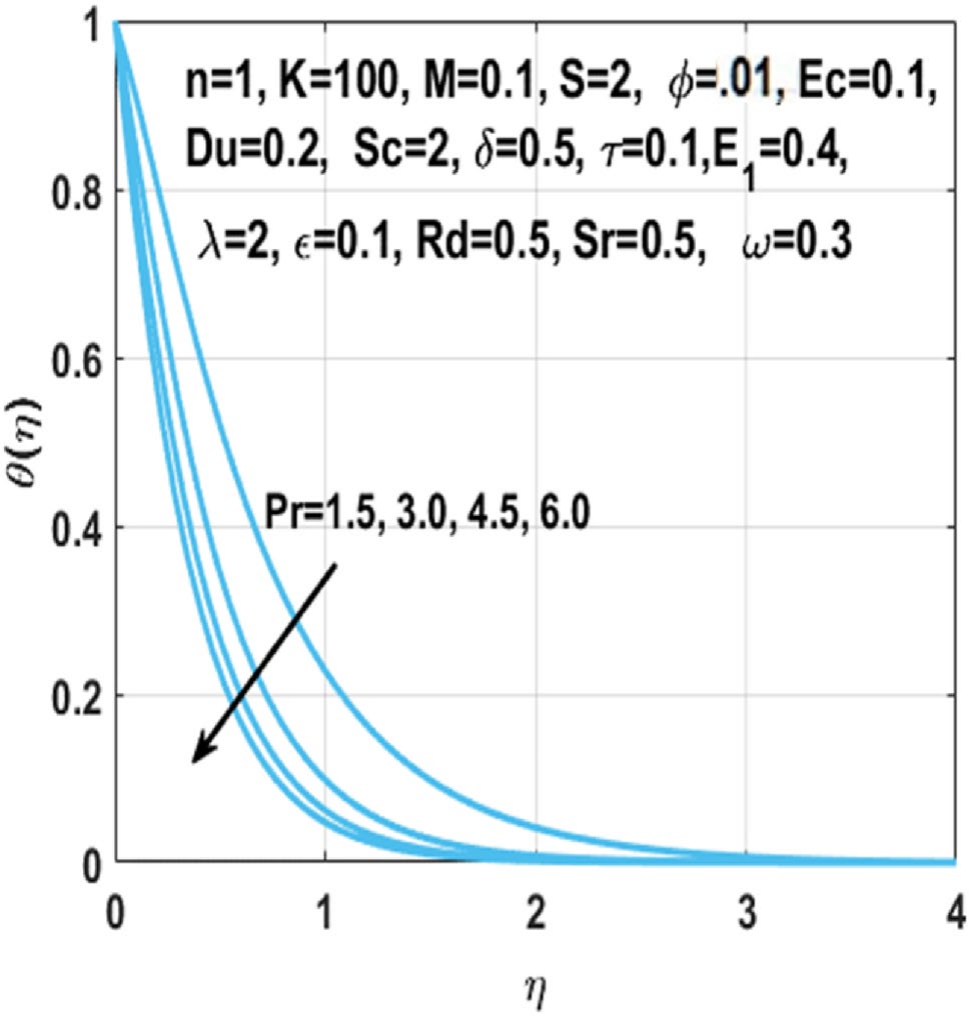
Impact of $Pr$ on $\theta \left( \eta \right)$.

The graphical representation of the concentration, temperature, velocity, Nusselt number, entropy generation, skin friction coefficient, Bejan number and Sherwood number are described in this section. Graphs of velocity with radial direction $f^{\prime } \left( \eta \right)$ are shown in Figs. 2 and 3 and they are similarly affected by both $K$ and $M$. Figure 2 shows that the curvature parameter $K$ is in direct relation with radius of the sheet resulting in less space for particles to stick when sheet radius decreases and because of that stretching rate decreases so the fluid velocity diminishes. Figure 3 explicated the decreasing effect of $M$ on velocity profile $f^{\prime } \left( \eta \right)$. As larger the Hartmann number $M$, the higher the resistive force due to that momentum boundary layer thickness is reduced. The decrease in nanoparticles volume fraction $\phi$ consequences the decrease in temperature profile $\theta \left( \eta \right)$ shown in Fig. 4, because with the higher value of $\phi$, more nanoparticles will be made available to conduct to the heat dissipated from the surface. Figure 5 shows accelerating behavior of $\theta \left( \eta \right)$ depending on the greater ${\text{Du}}$, there is an increase in temperature and thermal diffusion. The decreasing effect of ${\rm Pr}$ on $\theta \left( \eta \right)$ are shown in Fig. 6. Higher ${\text{Pr}}$ reduces the thickness of the thermal boundary layer and $\theta \left( \eta \right)$.because, by definition, ${\text{Pr}}$ is the “ratio of the momentum diffusivity and thermal diffusivity.”

**Figure 7:**
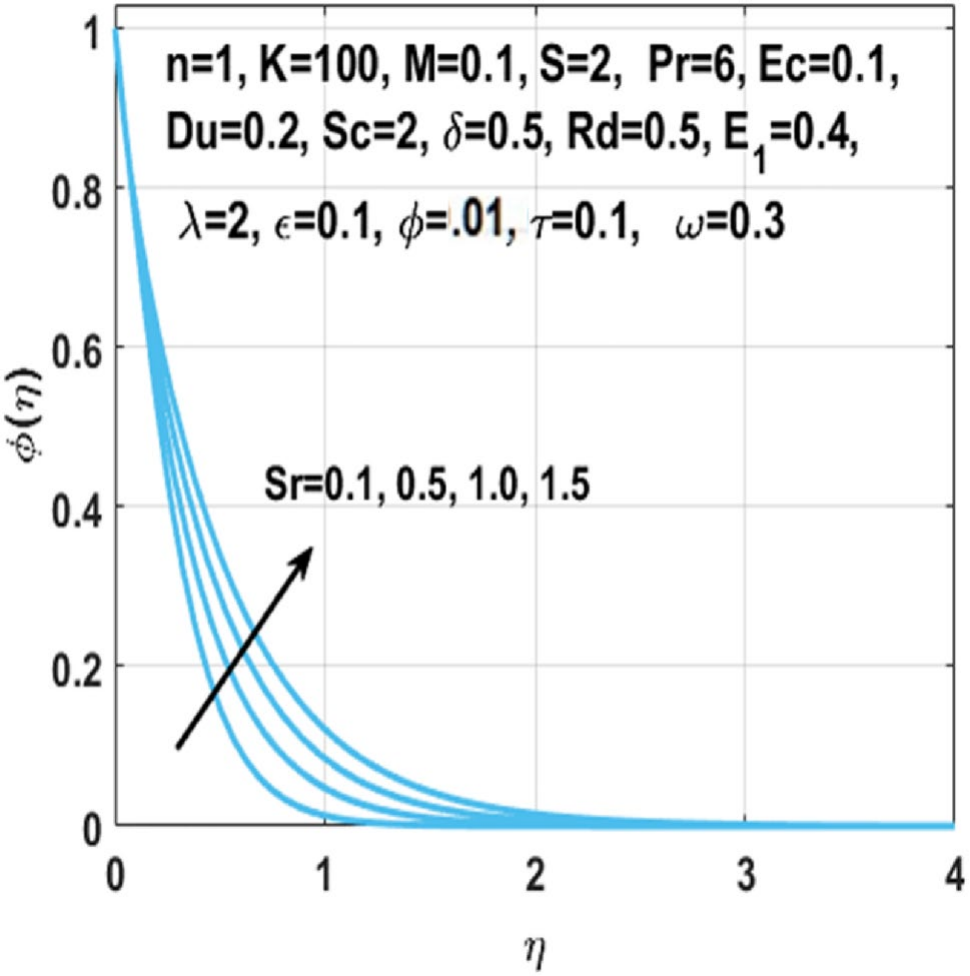
Impact of $Sr$ on $\phi \left( \eta \right)$.

**Figure 8:**
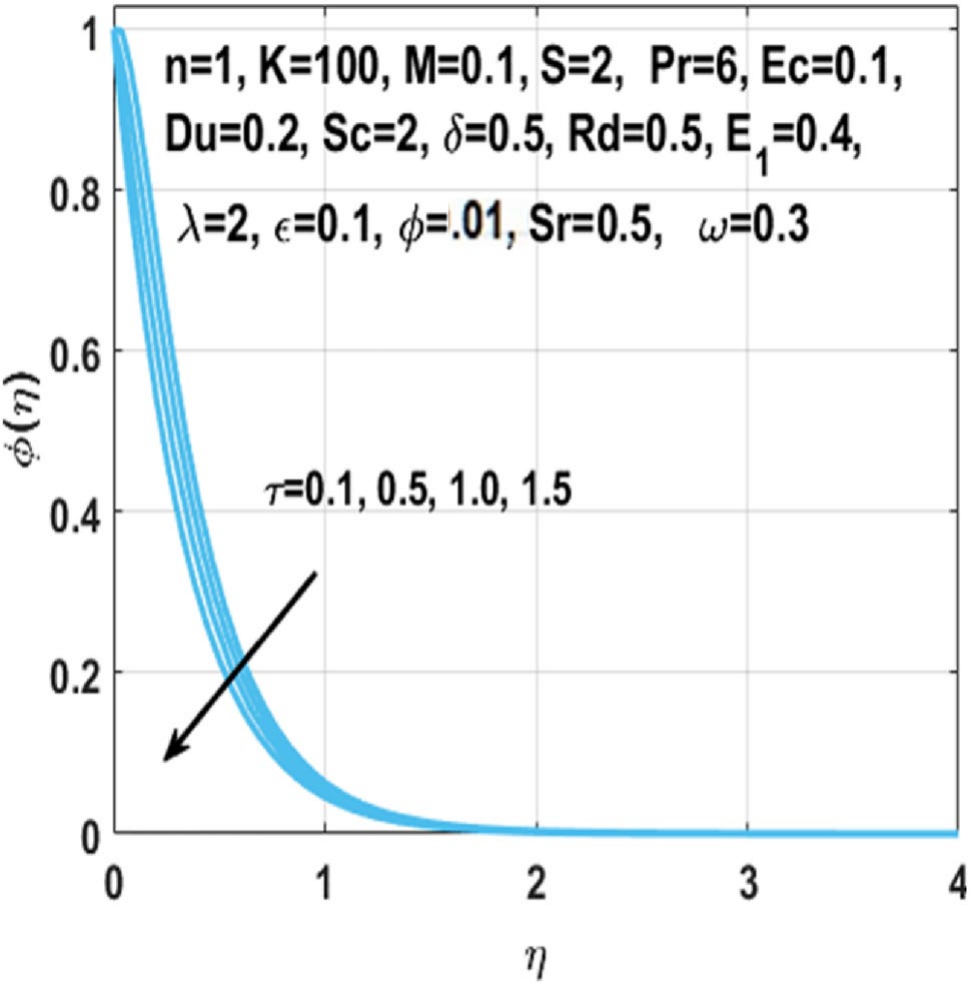
Impact of $\tau$ on $\phi \left( \eta \right)$.

**Figure 9:**
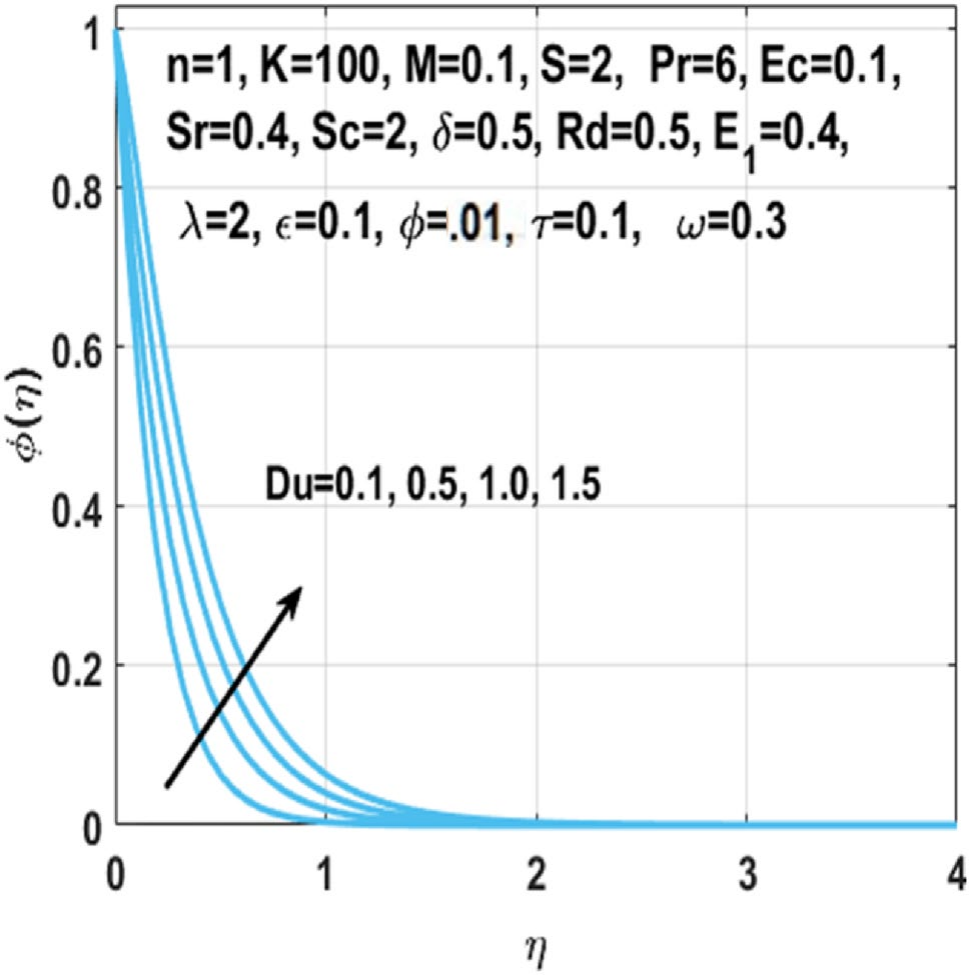
Impact of ${\text{Du }}$ on $\phi \left( \eta \right)$.

**Figure 10:**
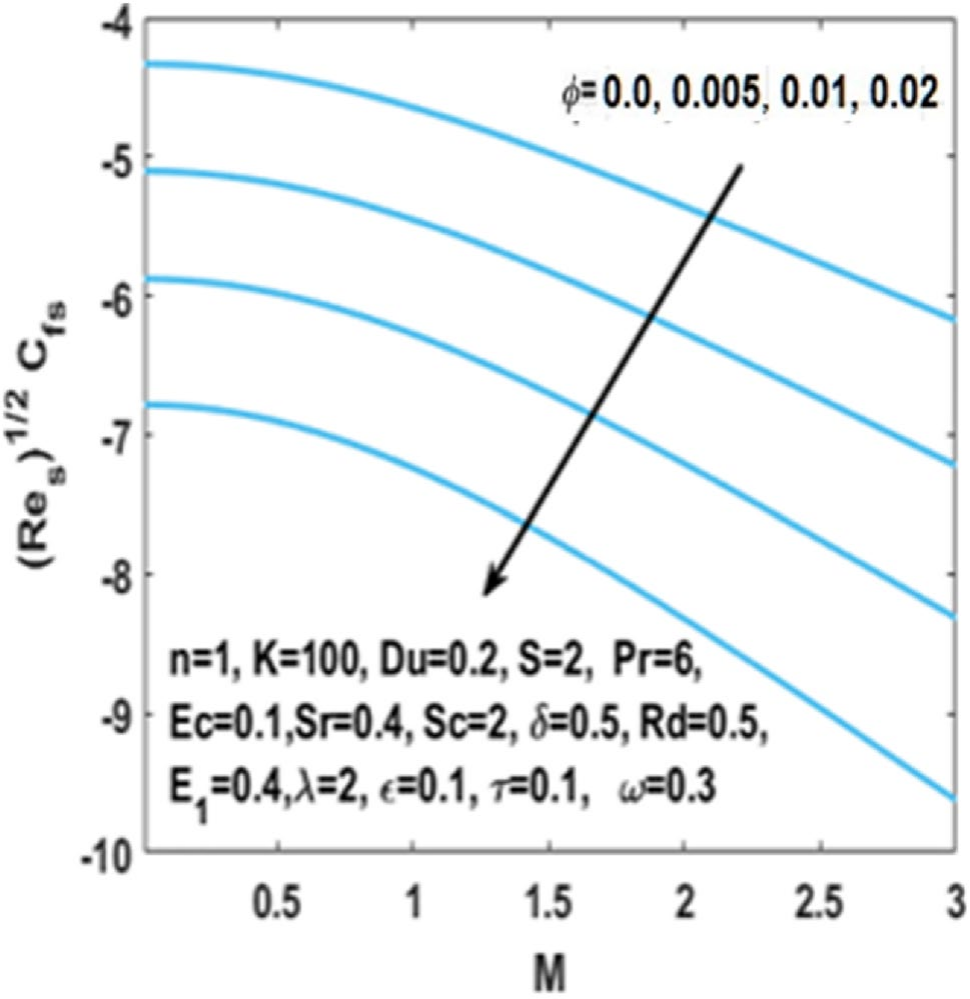
Impact of $\phi$ on skin friction w.r.t $M$.

**Figure 11:**
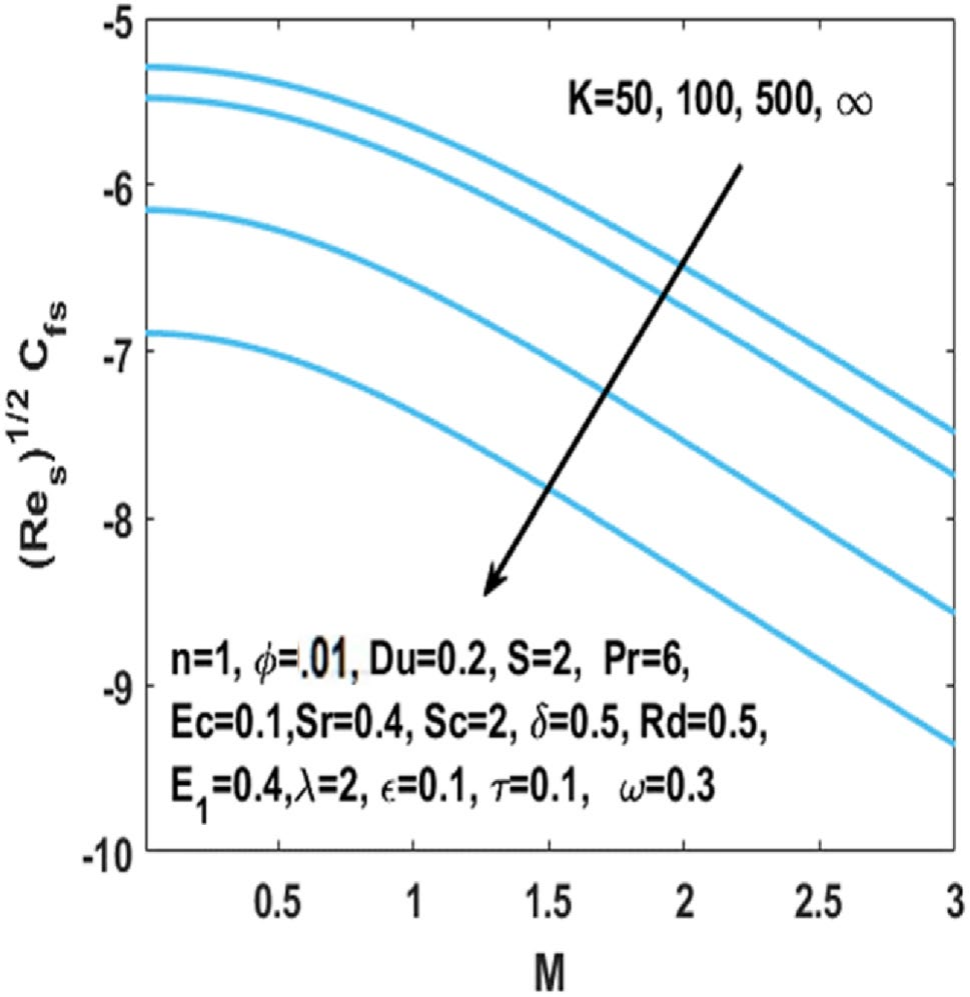
Impact of $K$ on skin friction w.r.t $M$.

From Figs. 7, 8 and 9 we can see that by the rising value of ${\text{Du}}$ ${\text{Sr}}$,τ and Du, the concentration profile $\phi \left( \eta \right)$ enhances respectively. By definition Soret number is the “effect resulting from the the proportion between temperature and concentration differences” and ${\text{Du}}$ is the “effect resulting from the proportion between concentration difference and temperature difference”. This clarifies that diffusive species having greater Soret and Dufour values results in higher concentration profile $\phi \left( \eta \right)$. The effect of chemical reaction parameter $\tau$ on the $\phi \left( \eta \right)$ is seen from Fig. 8. This figure depicts that the concentration profile is extremely dominated and decreases by the greater chemical reaction parameter while flowing in region. From Fig. 10, it is examined that the skin fraction coefficient $C_{fs}$ enhances with the larger nanoparticles volume fraction $\phi$. The reason behind this phenomenon is the higher density of nanofluid with nanoparticles fraction and higher density of nanofluid results in higher skin friction coefficient. Figure 11 illustrates the effect of $K$ on the coefficient of skin friction $C_{fs}$ through the Hartmann number $M$. Here, the curvature parameter $K$ and the magnitude of the skin friction coefficient are directly proportional to each other because when $K$ is larger, radius of surface decreases, it creates more resistance for fluid particles which leads to higher value of the skin friction coefficient $C_{fs}$.

**Figure 12:**
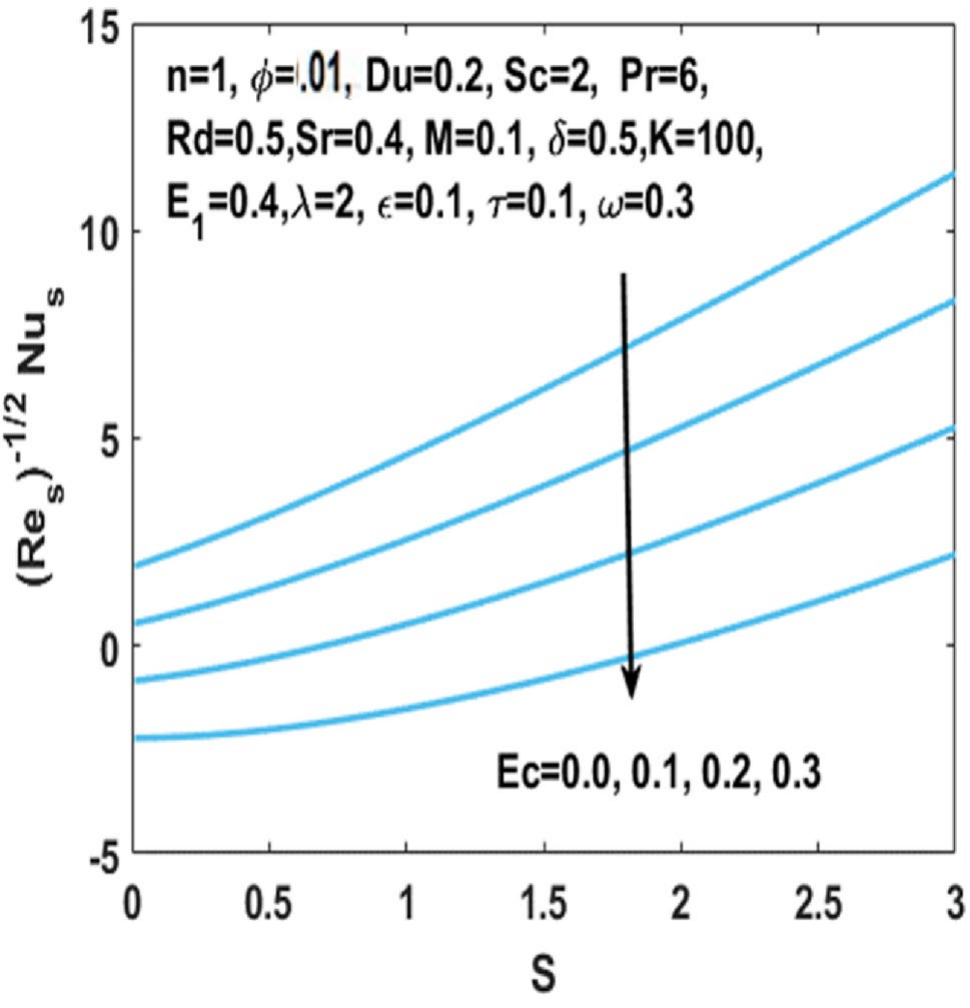
Impact of $Ec$ on Nusselt number w.r.t $S$.

**Figure 13:**
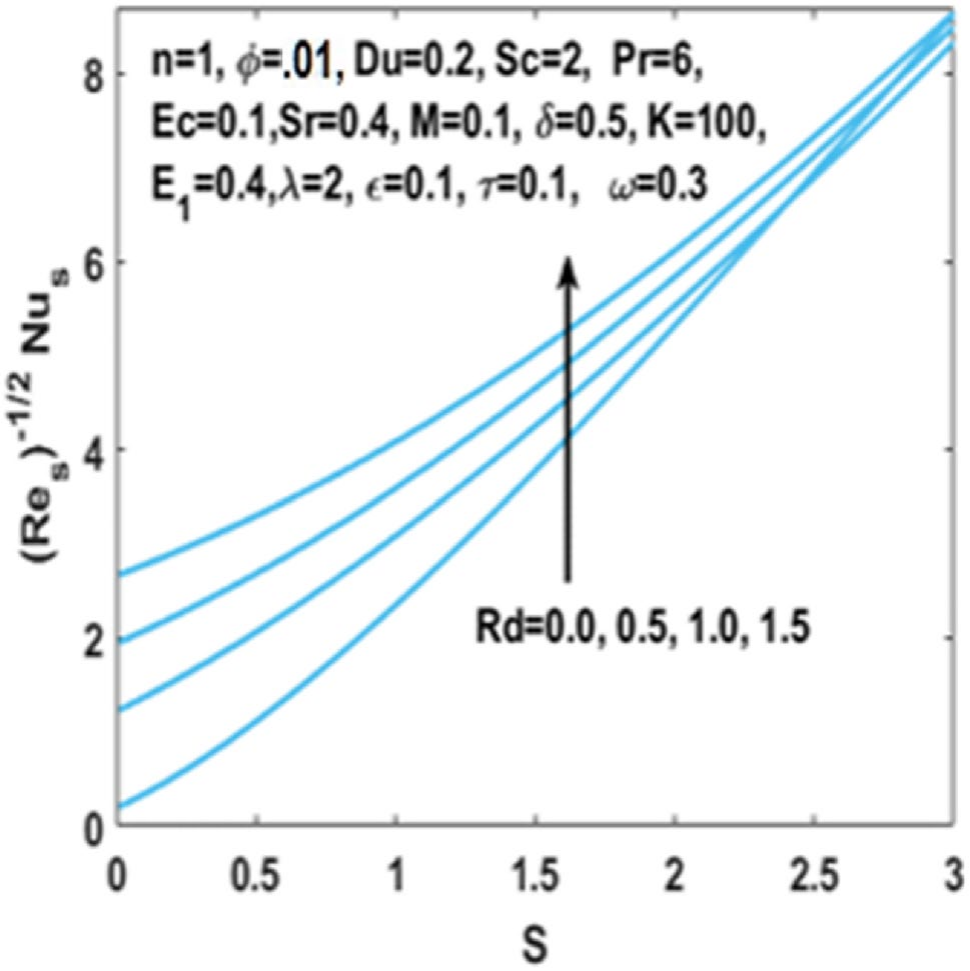
Impact of $Rd$ on Nusselt number w.r.t $S$.

**Figure 14:**
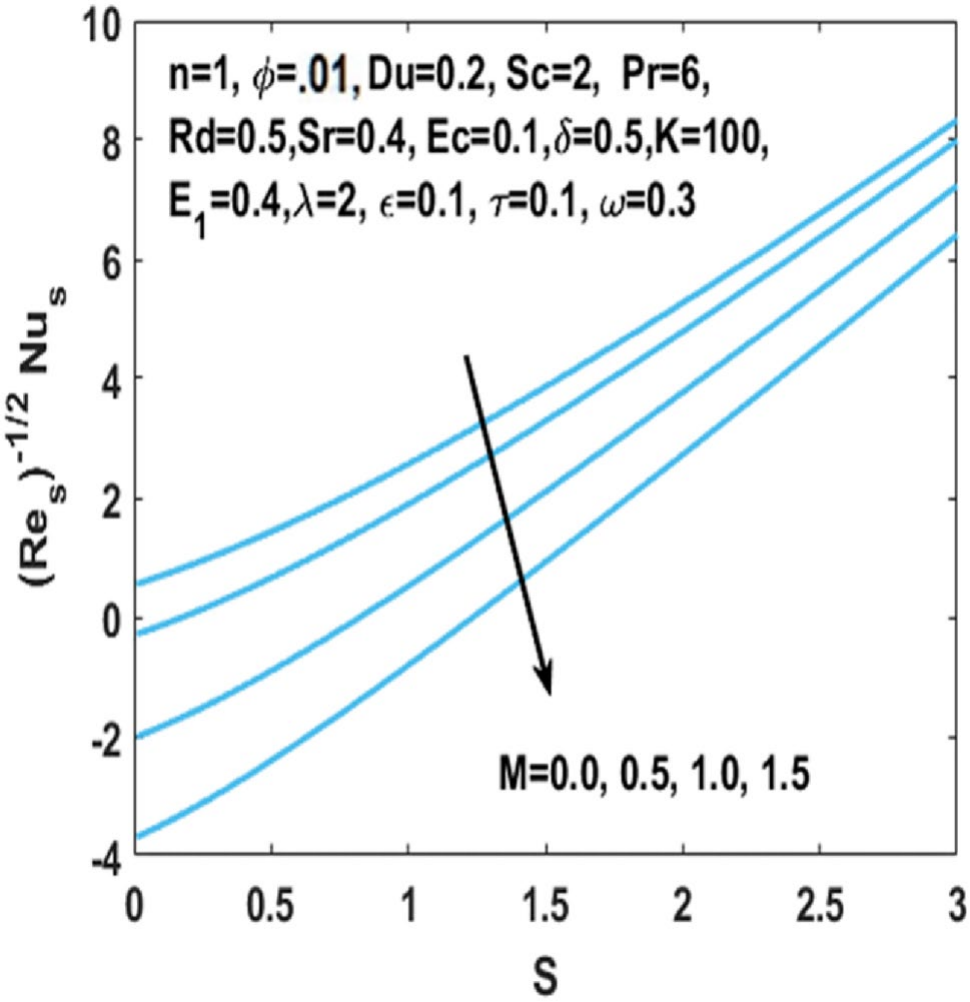
Impact of $M$ on Nusselt number w.r.t $S$.

**Figure 15:**
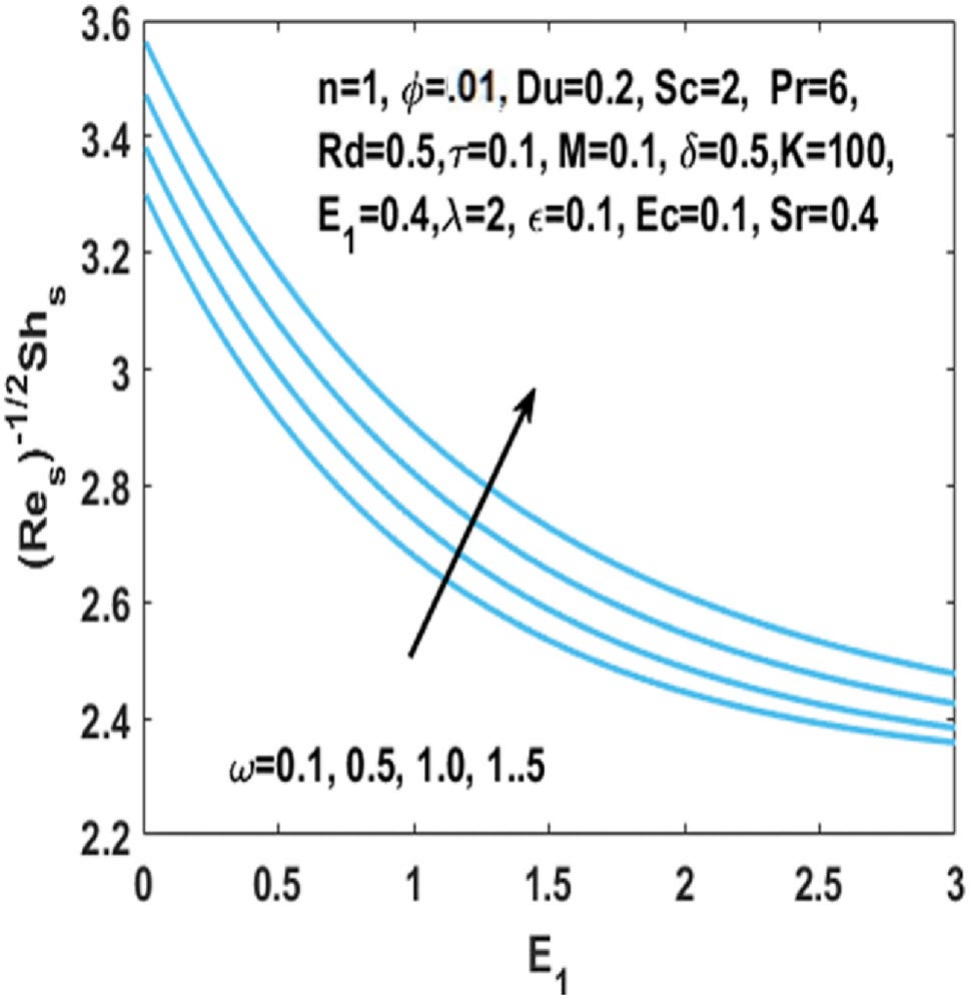
Impact of $\omega$ on Sherwood number w.r.t $E_{1}$.

**Figure 16:**
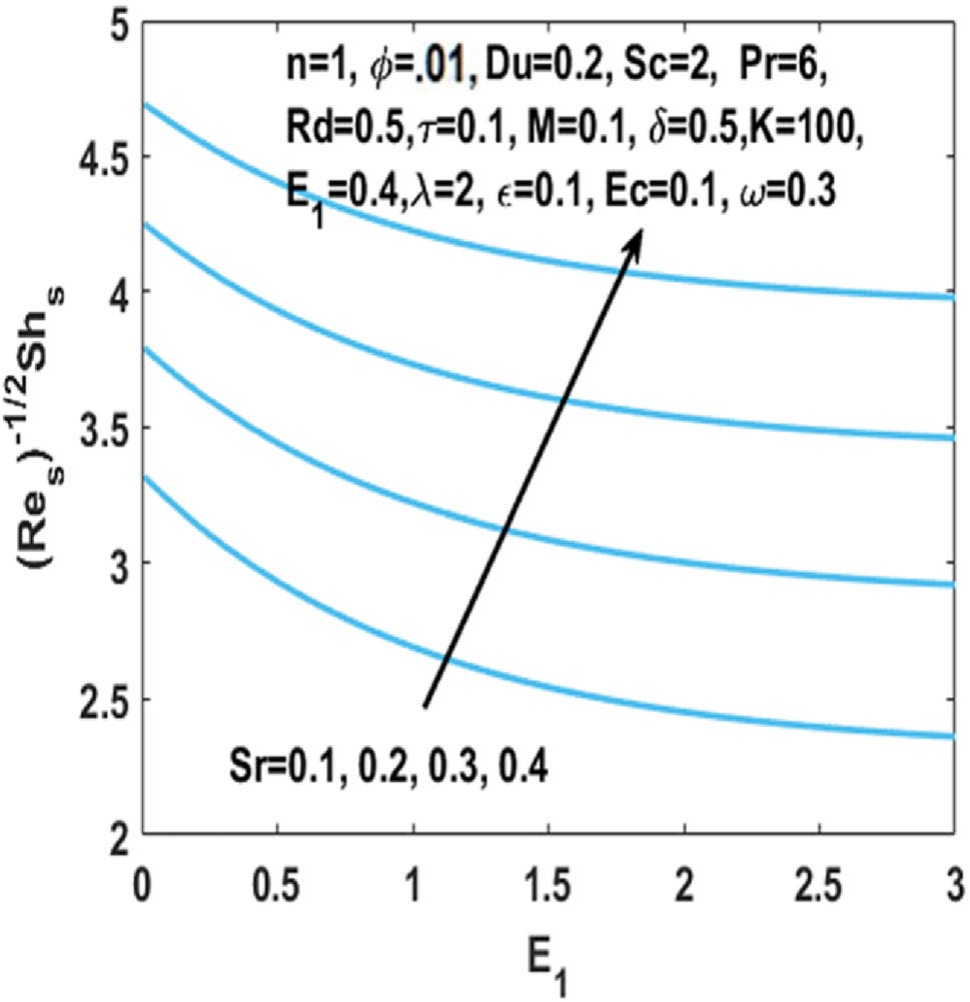
Impact of ${\text{Sr }}$ on Sherwood number w.r.t $E_{1}$.

**Figure 17:**
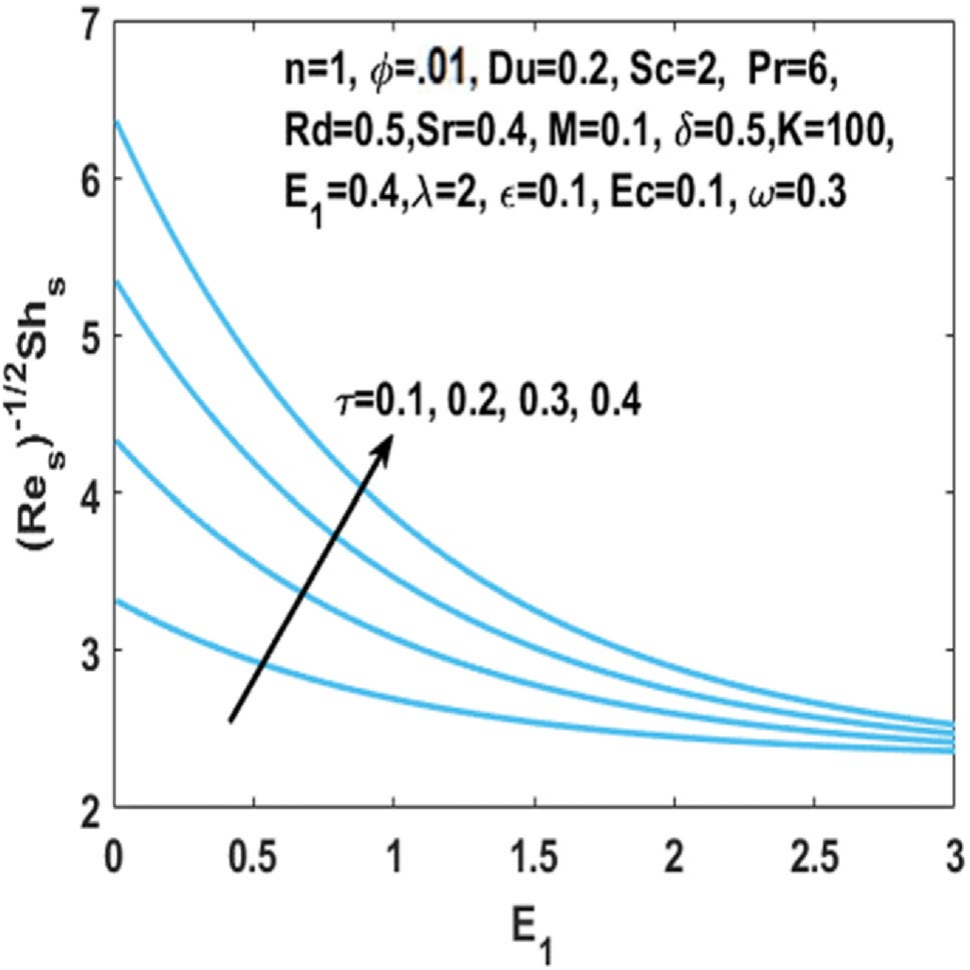
Impact of $\tau$ on Sherwood number w.r.t $E_{1}$.

In Fig. 12, we have analyzed the effects of suction and Eckert number on the ${\text{Nu}}_{s}$ which states that the Nusselt number ${\text{Nu}}_{s}$ is negatively connected to the Eckert number. Figures 13 and 14 discusses the relationship between the suction parameter $S$ and the change in magnitude of local Nusselt number. This is displayed on a graph for various radiation parameter values and Hartmann number $M$. These figures illustrate the indirect relationship among the Nusselt number and the radiation parameter $Rd$ as well as a reverse trend is observed for the Hartmann number $M$. Figures 15 and 16 show the effect of temperature difference parameter $\omega$ and Soret number $Sr$ on the Sherwood number ${\text{Sh}}_{s}$ versus activation energy $E_{1}$. As long as the temperature difference parameter $\omega$ and Soret number $Sr$ increases, the Sherwood number increases as well. The influence of chemical reaction parameter $\tau$ on the Sherwood number ${\text{Sh}}_{s}$ along with the activation energy $E_{1}$ is analyzed in Fig. 17. The Sherwood number rises because the chemical reaction parameter reduces the thickness of the concentration boundary layer.

**Figure 18:**
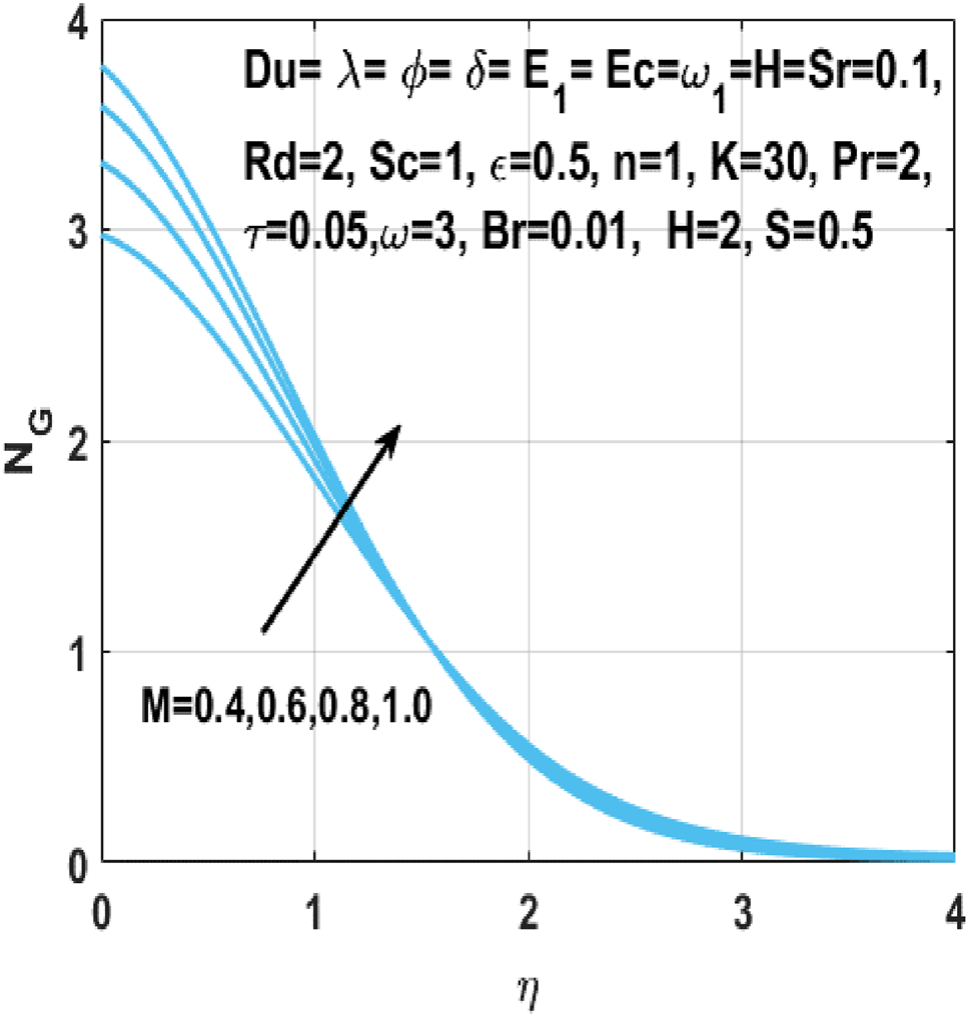
Impact of $M$ on $N_{G}$.

**Figure 19:**
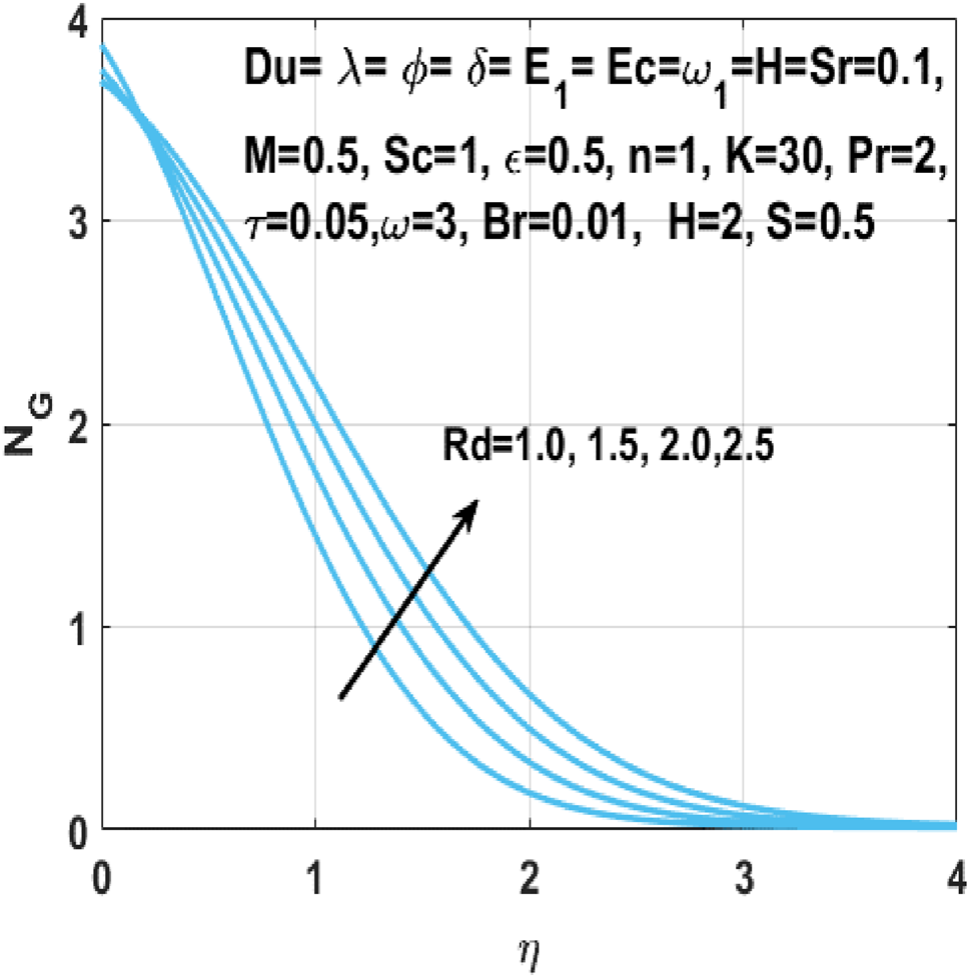
Impact of $Rd$ on $N_{G}$.

**Figure 20:**
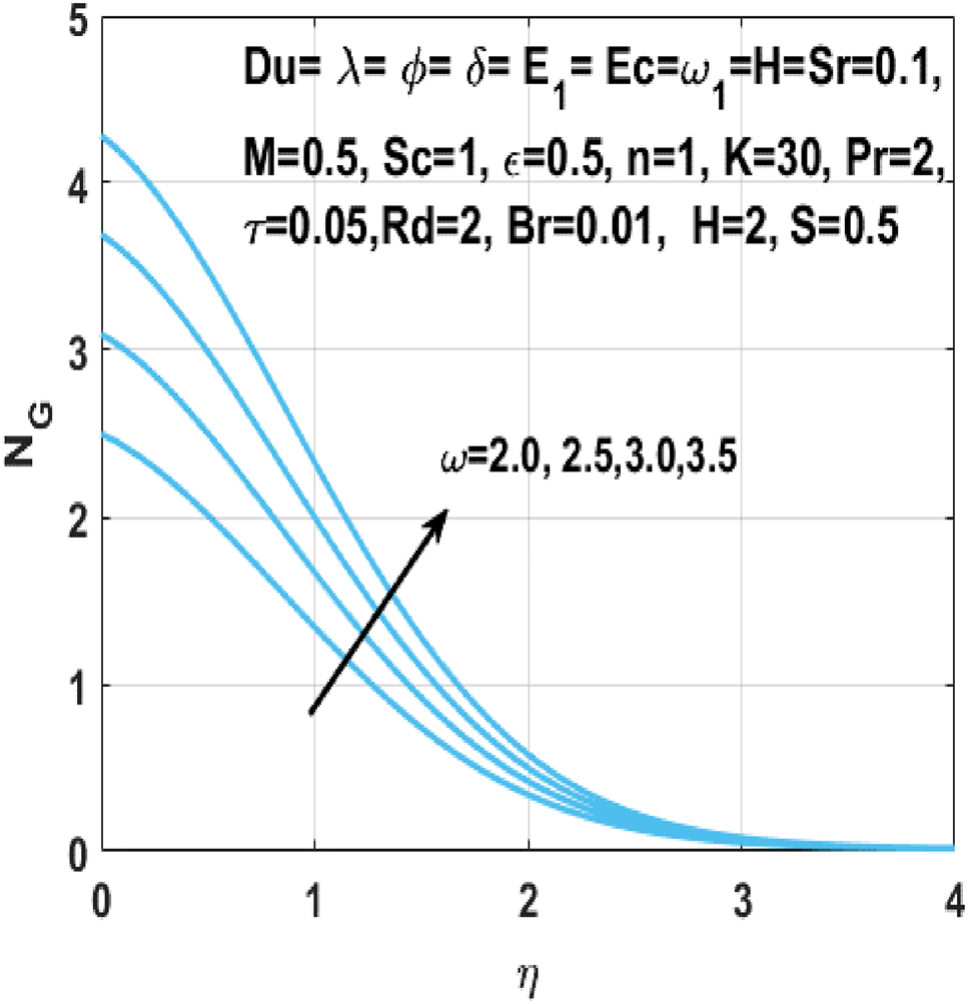
Impact of $\omega$ on $N_{G}$.

The Figs. 18 and 19 shows the enhancement of entropy generation $N_{G}$ by increase in Hartmann number $M$ and radiation parameter $Rd$ respectively. From these figures, it is seen that the entropy generation $N_{G}$ shows more noticeable increase within the existence of Hartmann number *M* and the radiation parameter *Rd*. Consequently, when magnetic field acts on the flow field, the fluid temperature rises because of the Lorentz forces. In addition, higher *Rd* results in higher temperature of fluid owing to growth in the movement of charged particles. Thus, an additional phenomenon viz vibration, internal displacement happens when temperature of fluid flow rises and results in boosting of entropy of the fluid flow system. Figure 20 shows the behavior of the rate of entropy generation $N_{G}$ against the temperature difference parameter $\omega$. Zhao et al.^29^ reported earlier that there is no objection to the existence of entropy, which specifies that the entropy generation $N_{G}$ is increasing with increasing values of temperature difference parameter $\omega$.

**Figure 21:**
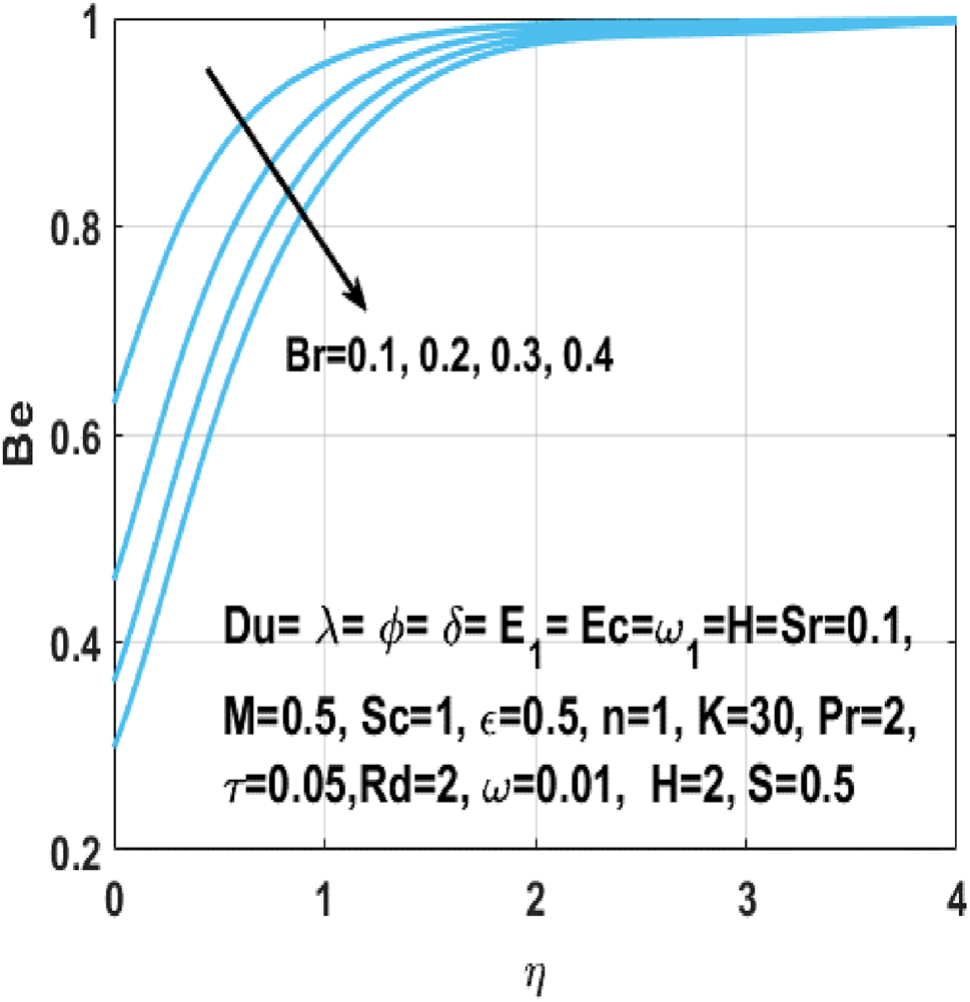
Impact of ${\text{Br }}$ on ${\text{Be}}$.

**Figure 22:**
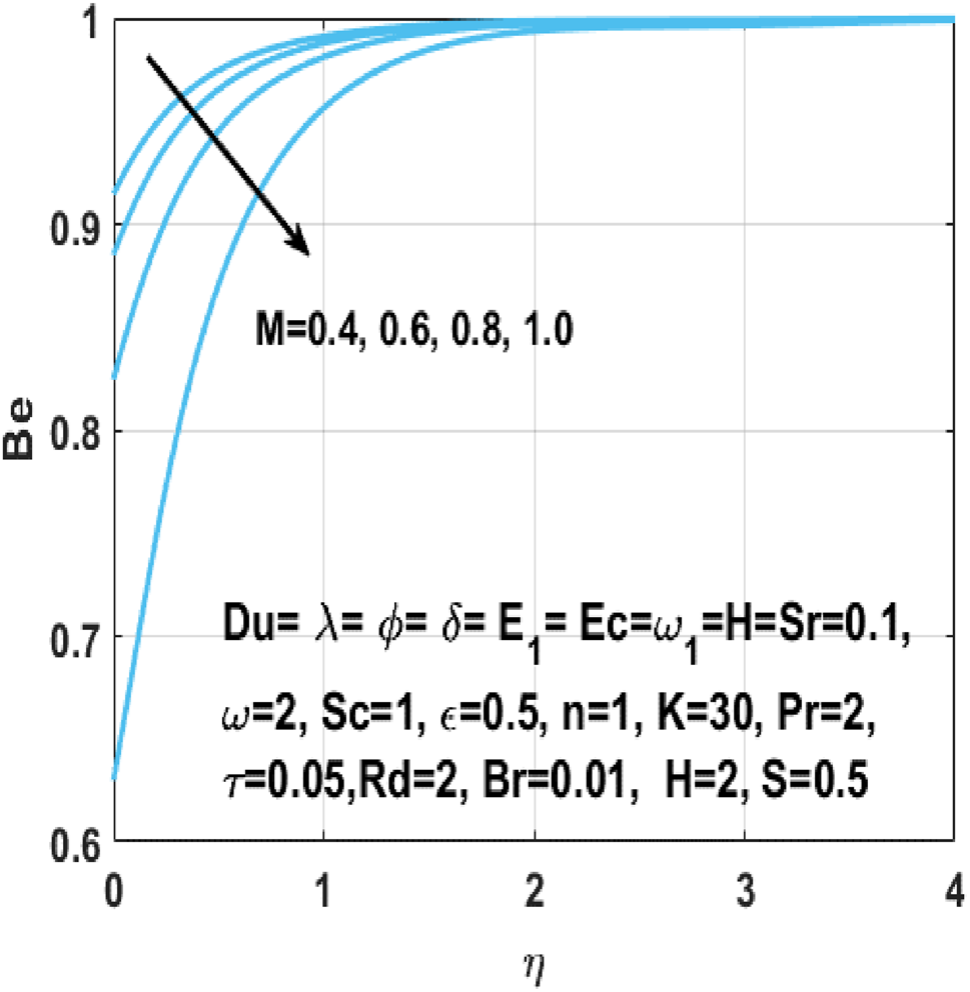
Impact of $M$ on ${\text{Be}}$.

The fluid temperature increases rapidly when a Lorentz force appears because of the magnetic field applied to the flow field. There is more viscous heating than heat transfer because of the conduction in the presence of larger Brinkman number $Br$, so resulting in high fluid temperature. The Figs. 21 and 22 explains the outcome of the Brinkman number ${\text{Br}}$ and the Hartmann number $M$ on the Bejan number ${\text{Be}}$. From these two scenarios we can see that these parameters negatively affect the Bejan number owning to the irreversibility of mass and heat transfer which is decreased by constant terms like fluid friction. It defines that the greater values of Brinkman number ${\text{Br}}$ effects having Joule heating and viscous dissipation are lesser than heat transfer irreversibility. Moreover, we examined the findings in Table 3 with the body of prior research to determine the validity of the study.

**Table 3:** Comparison of obtained results for $- f^{\prime \prime } \left( 0 \right) + \frac{{f^{\prime } \left( 0 \right)}}{K}$ with the existing literature for validation.

$K$	Sajid et al. ^50^	Abbas et al. ^51^	Present results	Error %
20	0.93561	1.03561	1.0332	0.2327
30	0.95686	1.02353	1.0223	0.1201
40	0.96759	1.01759	1.0182	0.0599
50	0.97405	1.01405	1.0141	0.0049
100	0.98704	1.00704	1.0071	0.00595
200	0.99356	1.00356	1.0035	0.0089

## Conclusion

The hydromagnetic stagnation flow of nanofluid under the radiation effect is analyzed by implementing the Soret Dufour model for heat and mass transport. Furthermore, bvp4c is employed to get the solution of the system of ordinary differential equations acquired by transforming the governing PDEs. The outcomes so acquired were related to the literature already in existence, and a reasonable degree of agreement was found, hence validating the solution. Some of the main outcomes that can be derived from this study are as follows:

- The velocity profile reduces for the larger curvature parameter $\left( K \right)$ and the Hartmann number $\left( M \right)$.
- The thermal and momentum boundary layer thickness enhances with the larger values of nanoparticles concentration $\left( \varphi \right)$.
- The Dufour number $\left( {{\text{Du}}} \right)$ and the Prandtl number $\left( {{\text{Pr}}} \right)$ have different impacts on the temperature profile.
- The Soret number $\left( {{\text{Sr}}} \right)$ and the Dufour number $\left( {{\text{Du}}} \right)$ steps up the concentration profile but chemical reaction rate parameter $\left( \tau \right)$ diminishes it.
- The nanoparticle concentration $\left( \varphi \right)$ and the curvature parameter $\left( K \right)$ minimizes the skin friction w.r.t. Hartmann number $\left( M \right)$.
- The Eckert number $\left( {{\text{Ec}}} \right)$ and the magnetic field parameter $\left( M \right)$ minimizes the Nusselt number but radiation parameter $\left( {Rd} \right)$ elevate the Nusselt number w.r.t. the suction $\left( S \right)$.
- The Hartmann number $\left( M \right)$, temperature difference parameter $(\omega$) and the radiation parameter $\left( {Rd} \right)$ steps up entropy generation $(N_{G} )$.
- The Bejan number is decreasing for larger values of Brinkman number $\left( {{\text{Br}}} \right)$ and Magnetic field parameter $\left( M \right)$.

## Data Availability

The corresponding author will provide the datasets used and/or analyzed during the current work upon reasonable request.

## List of symbols

*p*: Dimensional pressure (kg m^−1^ s^−2^)
$\mu_{f} , \mu_{s}$: Nano particles and base liquid dynamic viscosity (kg m^−1^ s^−2^)
$\mu_{nf}$: Nanofluid dynamic viscosity (kg m^−1^ s^−2^)
$\rho_{s} , \rho_{f }$: Nano particles and base liquid density (kg m^−3^)
$\rho_{nf}$: Nanofluids density (kg m^−3^)
$k_{f} , k_{s}$: Base liquid and nano particles thermal conductivity (W m^−1^ K^−1^)
$k_{nf}$: Nanofluids thermal conductivity (W m^−1^ K^−1^)
$\nu_{s} ,\nu_{f}$: Nano particles and base liquid kinematic viscosity (m^2^/s)
$\nu_{nf}$: Nanofluid kinematic viscosity
$\left( {\rho C_{p} } \right)_{f} , \left( {\rho C_{p} } \right)_{s}$: Base liquid and nanoparticles heat capacity (ML^2^/T^2^K)
$\left( {\rho C_{p} } \right)_{nf}$: Nanofluids heat capacity
$\sigma_{f} , \sigma_{s}$: Base liquid and nano particles electrical conductivity (S m^−1^)
$\sigma_{nf}$: Nanofluids electrical conductivity (S m^−1^)
$\alpha_{f} , \alpha_{s}$: Base liquid and nanoparticles thermal diffusivity (L^2^/T)
$\alpha_{nf}$: Nanofluids thermal diffusivity
$C_{{f_{s} }}$: Skin friction coefficient
$${\text{Sh}}_{s}$$: Sherwood number
$${\text{Nu}}_{s}$$: Nusselt’s number
$${\text{Re}}_{s}$$: Reynolds number
$T$: Temperature of the fluid (K)
$$\varphi$$: Nanoparticle’s concentration
$a$: Constant related to stretching/shrinking of the sheet
$s$: Arc length coordinate along the curved surface
$\tau_{rs}$: Wall shear stress
$\lambda$: Stretching/shrinking parameter
$B_{0}$: Magnetic field strength (T)
$r$: Normal to the tangent at any point of the curved surface
$u, v$: Velocity components in $s, r$ directions respectively (m s^−1^)
$R$: Curvature of the curves belt
$C$: Concentration of the fluid (mol m^−3^)
$C_{w} , C_{\infty }$: Concentration near and far away from the surface respectively
$T_{w} , T_{\infty }$: Temperatures near and far away from the surface respectively
$K$: Dimensionless curvature parameter
$M$: Magnetic field parameter
$$S$$: Suction parameter
$${\text{Ec}}$$: Eckert number
$$\delta$$: Porosity parameter
$${\text{Pr}}$$: Prandtl number
$j_{w}$: Wall heat flux
$q_{w}$: Wall heat flux
$$\nu_{w}$$: Suction velocity
$\sigma^{*}$: Stefan–Boltzmann constant (W m^−2^ K^−4^)
$k^{*}$: Mean absorption coefficient (m^−1^)
$k_{T}$: Thermal diffusion ratio
$$D_{m}$$: Molecular diffusivity (m^2^ s^−1^)
$c_{s}$: Concentration susceptibility (Kg m^−3^)
$k_{1}$: Boltzmann constant (8.314 J/K mol)
$k_{r}$: Chemical reaction rate (mol L^−1^ s^−2^)
$n$: Fitted rate constant (W m^−2^ K^−1^)
$E_{a}$: Activation energy (KJ mol^−1^)
$$P$$: Dimensionless pressure
$$f$$: Stream function
$$\eta$$: Similarity variable
$$f^{\prime}$$: Dimensionless velocity
$\theta$: Dimensionless temperature of fluid
$\phi$: Dimensionless concentration of fluid
$\omega$: Temperature difference parameter
$${\text{Br}}$$: Brinkman number
$\tau$: Dimensionless chemical reaction rate parameter
$$Rd$$: Radiation parameter
$${\text{Du}}$$: Dufour number
$${\text{Sc}}$$: Schmidt number
$E_{1}$: Dimensionless activation energy parameter
$$L$$: Slip length
$\epsilon$: Dimensionless slip length
$$H$$: Diffusion parameter
$$\omega_{1}$$: Concentration difference
$N_{G}$: Local entropy generation
$K_{p}$: Permeability of porous medium
$${\text{Sr}}$$: Soret number
